# Radioiodination of Two Carborane‐Based Dual Cyclooxygenase‐2/5‐Lipoxygenase Inhibitors and Their In Vitro and In Vivo Evaluation

**DOI:** 10.1002/cbic.202600004

**Published:** 2026-03-31

**Authors:** Jonas Schädlich, Martin Ullrich, Cathleen Haase‐Kohn, Robert Wodtke, Sebastian Braun, Maximilian Molitor, Bettina Hofmann, Dieter Steinhilber, Klaus Kopka, Evamarie Hey‐Hawkins, Jens Pietzsch, Markus Laube

**Affiliations:** ^1^ Institute of Radiopharmaceutical Cancer Research Helmholtz‐Zentrum Dresden‐Rossendorf Dresden Germany; ^2^ Faculty of Chemistry and Food Chemistry School of Science Technische Universität Dresden Dresden Germany; ^3^ Institute of Bioanalytical Chemistry Centre for Biotechnology and Biomedicine (BBZ) Faculty of Chemistry and Mineralogy Leipzig University Leipzig Germany; ^4^ Institute of Pharmaceutical Chemistry Johann Wolfgang Goethe‐Universität Frankfurt Frankfurt Germany; ^5^ Faculty of Chemistry and Chemical Engineering Department of Chemistry Babeș‐Bolyai University Cluj‐Napoca Romania

**Keywords:** carboranes, dual inhibition, eicosanoids, inflammation, single‐photon emission computed tomography

## Abstract

Cyclooxygenase‐2 (COX‐2) and 5‐lipoxygenase (5‐LO) are key enzymes in prostanoid and leukotriene signaling and are overexpressed in various cancers, correlating with malignancy and metastasis. This renders them promising molecular targets for radiotracer development. In this study, isomeric *closo*‐dicarbadodecaborane(12)‐based dual COX‐2/5‐LO inhibitors (**1** and **2**) served as scaffolds for iodine‐123‐labeling. Iodinated derivatives **3** and **4** inhibited 5‐LO more potently than COX‐2 in vitro (*IC*_50_ 0.62 µM and 41.3 µM (**3**); 0.54 µM and 67.7 µM (**4**)). Radioiodination yielded 39%–71% of **[** ^**123**^ **I]3** and **[** ^**123**^ **I]4**; and the formulations were stabilized with antioxidants. Cellular uptake of **[** ^**123**^ **I]3** and **[** ^**123**^ **I]4** was evaluated in human cells with distinct COX‐2 and 5‐LO expression: U87 glioblastoma, HT‐29 colorectal carcinoma cells, THP‐1 monocytes (MC) and macrophages. Consistent with the observed inhibitory potency, the uptake of the radiotracers proved to be independent of COX‐2 expression. In contrast, it was influenced by 5‐LO expression in tumor but not inflammatory cells. Single‐photon emission computed tomography imaging in tumor xenograft mouse models revealed no tumor retention of **[** ^**123**^ **I]3** and **[** ^**123**^ **I]4** due to rapid metabolism via radiodeiodination. Despite successful radiosynthesis and in vitro evaluation of iodine‐123‐labeled COX‐2/5‐LO inhibitors, improved inhibitory activity and stability are required for development of suitable radiotracers.

## Graphical abstract

Two dicarbadodecaborane(12)‐based dual cyclooxygenase‐2/5‐lipoxygenase (COX‐2/5‐LO) inhibitors were ^123^I‐labeled. Refinement of labeling and formulation conditions and extensive in vitro characterization are presented. Cell uptake studies in COX‐2‐ and 5‐LO‐overexpressing cell lines showed COX‐2‐independent and partly 5‐LO‐dependent uptake. However, limited in vivo stability and lack of uptake in tumor xenografts preclude further development.

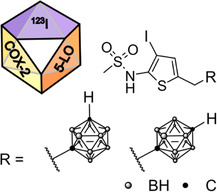

## 1 Introduction

The worldwide impact of cancer persists as a prominent global challenge for the medical sector, which requires diagnostic tools for early diagnosis, staging, and targeted therapeutic intervention. In vivo molecular imaging modalities, such as positron emission tomography and single‐photon emission computed tomography (SPECT), are commonly utilized in clinical practice for the noninvasive visualization and characterization of tumor biology [1, 2]. Cyclooxygenase‐2 (COX‐2) and 5‐lipoxygenase (5‐LO) are key enzymes in eicosanoid signaling pathways during inflammatory processes [3, 4, 5, 6]. Both enzymes play critical roles in cancer development, metastasis, resistance, and therapy [7, 8, 9, 10]. The multitude of relevant oncological functions of COX‐2 and 5‐LO highlights their potential as molecular targets for radio‐tracer development.

The isozymes COX‐1 and COX‐2 catalyze the conversion of arachidonic acid to the common prostaglandin progenitor PGH_2_. COX‐1, which is expressed in various tissues, is responsible for the biosynthesis of prostanoids, which regulate thrombocyte aggregation, gastric acid, mucus, and kidney function [3, 4, 8]. Conversely, COX‐2 is typically expressed by only a few cell types but is inducible by various stimuli during pain and inflammatory processes. COX‐2‐derived PGE_2_ plays a pivotal role in potentiation of pain and induction of fever [3, 4]. This plethora of physiological functions is contrasted by the involvement of prostanoids in tumorigenesis and the progression of cancer, including metastasis [7, 8, 9, 11]. Several cancer entities show overexpression of COX‐2 (e.g. colorectal adenocarcinoma or glioblastoma multiforme) [12, 13]. Furthermore, a poor prognosis and resistance to chemo‐ and radiotherapy are correlated with COX‐2 expression [7, 14, 15]. In line with these findings, application of unselective COX inhibitors (nonsteroidal anti‐inflammatory drugs, NSAIDs) was found to be associated with a reduced cancer risk [16, 17, 18]. Clinical studies also demonstrated that celecoxib is effective as a chemopreventive, a (neo‐)adjuvant agent during chemotherapy, or a radiosensitizer in several types of cancer [19, 20, 21, 22, 23, 24]. Nevertheless, patients’ responses toward the (co‐)application of coxibs (selective COX‐2 inhibitors) have varied significantly [25, 26], therefore necessitating further studies for efficient patient stratification for coxib indications. Ideally, a radiotracer visualizing the COX‐2 expression in the tumor tissue would substantially aid this process.

Leukotrienes exert a wide range of signaling functions such as chemotaxis, induction of pro‐inflammatory mediators, smooth muscle contraction, and regulation of endothelial barrier function [3, 5, 6]. The mediators originate from the progenitor leukotriene A_4_ (LTA_4_), which is synthesized from arachidonic acid by 5‐LO [3, 6, 27]. The enzyme is mainly expressed by leukocytes, macrophages, and dendritic cells [5]. Therefore, aberrant expression of 5‐LO in nonimmune cells is primarily associated with carcinogenesis and correlates with poor prognosis in cancers such as colorectal, esophageal, and prostate cancer, and glioblastoma [10, 28, 29, 30, 31]. In this context, the coexpression of 5‐LO and COX‐2 in glioblastoma was associated with a lower overall survival than high expression of either enzyme alone [32]. The mechanisms by which 5‐LO acts as a growth promoter in solid tumors, rather than supporting host defense and tumor suppression, appear to cover a combination of pathways. In contrast, the role of 5‐LO in hematological malignancies (such as myeloid leukemia) is heterogeneous and not consistently predictive of the prognosis [27]. In vitro studies have demonstrated anti‐proliferative and pro‐apoptotic activity of selected 5‐LO inhibitors in various cancer cell lines [33, 34, 35]. Furthermore, 5‐LO inhibitor zileuton in combination with the tyrosine kinase inhibitor imatinib has entered phase I clinical trial for treatment of BCR‐ABL^+^ CML, following the discovery that 5‐LO activity is required for the self‐renewal of leukemic stem cells [27, 36, 37].

With arachidonic acid as the common substrate of 5‐LO and COX‐2, the inhibition of the latter results in a surge of leukotrienes. This metabolic shunting contributes to the class side effects observed during therapy with NSAIDs or coxibs, such as gastric ulcers, exacerbation of respiratory diseases, and cardiovascular events. Furthermore, the leukotriene overproduction following COX‐2 inhibition was found to impede the anti‐cancer efficacy in colorectal adenocarcinoma cells in vitro [38]. A similar effect of substrate shunting is not characterized to the same extent for 5‐LO inhibition. 5‐LO inhibitors are less extensively studied and suffer from other dose‐limiting effects than prostanoid surge. However, an increased prostaglandin release in 5‐LO‐deficient mice suggests that a shift toward prostanoid production under 5‐LO inhibition is likely [10, 39]. Therefore, the combined inhibition of COX‐2 and 5‐LO is highlighted as a strategy for the development of safer NSAIDs [40, 41, 42].

In vitro results support the use of dual (heterobivalent) COX‐2/5‐LO inhibitors or combinations of selective inhibitors in cancer therapy. Cytotoxic, anti‐metastatic, and radiosensitization effects have been reported toward various cancer cell lines [43, 44, 45, 46, 47]. In the search for dual inhibitors, NSAIDs exerting 5‐LO inhibition were investigated as pharmacophores (e.g. sulindac, tebufelone), while alternative approaches focused on a structural combination of COX and 5‐LO inhibitors, leading to a small library of structurally diverse compounds [48, 49, 50]. Accordingly, Navarrete et al. demonstrated that the presence of a catechol and a benzenesulfonamide moiety adjacent to a pyrazoline core leads to potent COX‐2/5‐LO inhibitors, which may be modifiable with organometallic motives [47]. Additionally, a 5‐LO inhibitor developed by Beers et al. (2‐phenoxythiophene derivative RWJ‐63 556) was later demonstrated to act as a COX‐2 inhibitor [51, 52]. Recently, our group presented potent COX‐2/5‐LO inhibitors carrying a *closo*‐dicarbadodecaborane(12) moiety using this scaffold [53].

Carboranes (*closo*‐dicarbadodecaboranes(12)) have gained interest as pharmacophores for their chemical and biological stability. The clusters consist of ten boron and two carbon vertices forming an icosahedral cage. Analogous to substitution nomenclature for benzene rings, the three isomers of carboranes are called *ortho*, *meta*, and *para* according to the relative positions of the carbon vertices. Carboranes slightly outsize a rotating benzene, which renders them promising phenyl mimetics. The unsubstituted clusters are highly lipophilic; however, this property may be modified by the removal of one boron vertex from the *closo* cage, resulting in the formation of an anionic *nido* species. Furthermore, carboranes can be derivatized via the two carbon vertices but also via several boron vertices [54, 55].

Despite intensive research during the past two decades, the limitations of COX‐2 imaging agents persist due to off‐target effects. Typically, high lipophilicity leads to reduced tumor accumulation and metabolic instability [56, 57]. The sulfones [^18^F]Triacoxib and [^18^F]pyricoxib possess high COX‐2 selectivity and are less prone to off‐target interactions than sulfonamides. However, both tracers exhibit only partly specific cell and tumor uptake [58, 59]. Our previous work focused on the development of COX‐2‐targeting radiotracers by variation of the aromatic backbone and ^11^C‐ and ^18^F‐labeling [60, 61, 62]. Additionally, we performed studies addressing the oxidative metabolism of radiofluorinated COX‐2 inhibitors, and explored potential opportunities for their stabilization [63, 64]. After demonstrating selective uptake in a neuroinflammation model, pyrimidine‐based COX‐2 inhibitor [^11^C]MC1 is currently enrolled in clinical trials for diagnosis and characterization of (neuro)inflammatory diseases [65]. Thus far, the repurposing of [^11^C]MC1 for tumor imaging has demonstrated promising preclinical results in colorectal cancer models [66]. Notably, the MC1 derivative BRD1158 offers faster COX‐2 binding kinetics and could further improve detection [67]. In contrast to 12‐LO and FLAP, no 5‐LO targeting radiotracers have been reported so far [68, 69]. We hypothesize that dual radiotracers hold great potential for visualizing both COX‐2‐ or 5‐LO‐positive tumor tissue and the presence of COX‐2 or 5‐LO expressing immune cells in the tumor microenvironment (TME). The selective blocking of either enzyme prior to tracer administration could permit differentiation of the two targets, allowing for a more detailed characterization of the tumor entity and TME. In radionuclide therapy, on the contrary, dual targeting possesses the chance of increased accumulation in tumor and TME, which could improve therapy outcomes.

The aim of this study was the development of imaging agents which circumvent the current limitations of COX‐2 radiotracers and serve as the first 5‐LO targeting radiotracers. Accordingly, we have harnessed the metabolic stability of the carborane moiety to increase the biological half‐life of the radiotracers. Iodine‐123 (t_1/2_ = 13.2 h) was selected as a suitable radionuclide for visualization over several hours, accommodating for the relatively slow kinetics of intracellular accumulation and time‐dependent inhibition observed for COX‐2 inhibitors [70]. Moreover, selective blocking experiments in vitro and in vivo were carried out to evaluate target selectivity. Furthermore, analysis of radiolabeled metabolites was undertaken to elucidate the metabolic pathways of carboranes, due to their expanding applications in medicinal chemistry.

## 2 Results and Discussion

### 2.1 Chemical Synthesis

**SCHEME 1:**
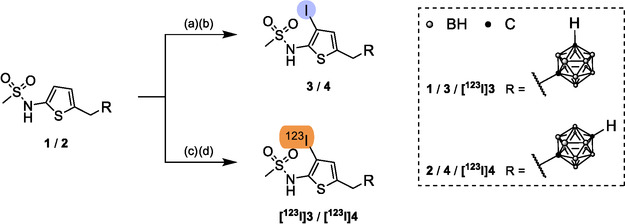
Iodination and radioiodination of **1** and **2**. (a) 1.1 molar equivalents (eq) NaI, 1.1 eq CAT, 30% v/v DMSO/H_2_O, rt (18–20°C), 60–75 min. (b) 20 eq Na_2_S_2_O_5_. (c) [^123^I]NaI, 1 eq CAT, 4% v/v DMSO/H_2_O, rt, 10 min. **1** and **2** were synthesized as previously described [53].

The synthesis of the reference iodo‐compounds **3** and **4** started from **1** and **2**, respectively. We recently reported already on their syntheses and characterization as derivatives of the dual COX‐2/5‐LO inhibitor RWJ‐63 556 [53]. Iodination of **1** and **2** was achieved under reaction conditions adjusted from standard electrophilic radioiodination techniques using chloramine‐T (CAT, Scheme 1). The iodinated compounds **3** and **4** were obtained in microscale experiments in 60% and 14% yields, respectively, and gave the chlorinated byproducts Cl‐**1** and Cl‐**2** (not isolated, UPLC‐MS, Figures S 1–3, Supporting Information (SI)). ^1^H and ^11^B{^1^H} NMR spectroscopy confirmed that the thiophene moiety was iodinated. This finding is consistent with the electron‐rich nature of thiophene and the comparatively lower reactivity of the *meta*‐ and *para*‐*closo*‐carborane clusters under electrophilic iodination conditions [71, 72]. NMR spectroscopy revealed that the *C*−3 carbon adjacent to the methylsulfamoyl substituent of the thiophene ring was iodinated (Figures S 4–11, S 12–19, SI). HRMS spectra of **3** and **4** were in accordance with the expected isotopic distribution (Figures S 21 and S 23, SI).

### 2.2 COX Inhibition

The inhibitory potency of **3** and **4** toward ovine COX‐1 and human recombinant COX‐2 was determined with the COX Fluorescence Inhibitor Screening Assay Kit (Cayman Chemical Company). Celecoxib and SC‐560 served as references. Compounds **3** and **4** showed partial inhibition of COX‐1 and COX‐2 at concentrations of 100 µM, with subsequent *IC*_50_ determination providing values of 41.3 and 67.7 µM, respectively, for COX‐2 (Table 1). For both iodinated compounds, inhibitory potency was found to be lower compared to their respective nonhalogenated counterparts. This trend was additionally observed for alternative iodinated COX inhibitors [73, 74, 75]. It can be assumed that the exchange of a hydrogen atom for the spacious iodine substituent is unfavorable regarding the binding of the inhibitors within the substrate channel of the COX active site. The slight isozyme preference for COX‐2 which was observed for **1** and **2** is maintained after iodination. Additionally, the *para* isomers **2** and **4** showed lower potency for the inhibition of COX‐2 compared to the corresponding *meta* isomers **1** and **3**. Regarding the use of **3** and **4** as potential radiotracers, it must be highlighted that selective uptake into COX‐2 positive tumors of these moderately potent COX‐2 inhibitors may be limited.

**TABLE 1:** COX and 5‐LO inhibitory potential of **3** and **4** compared to precursors **1** and **2**, as well as references celecoxib, SC560 and BWA4C.

	Fluorescence‐based COX assay					Intact cell 5‐LO assay	
Compound	% Inhibition at 100 µM		*IC*_50_, µM		SI^a^	% Residual activity at 0.1 µM	*IC*_50_, µM
	COX‐1	COX‐2	COX‐1	COX‐2			
**1** b	/	/	18.1	4.7	3.8	30	c
**3**	35	53	>100^d^	41.3	>2.4	90	0.62
**2** b	/	/	24.5	6.0	4.1	31	c
**4**	33	37	>100^d^	67.7	>1.4	93	0.54
**GA**	55	89	73	2.4	30	/	/
**Celecoxib**	/	/	/	0.078^e^	/	/	/
**SC‐560**	/	/	0.008^f^	/	/	/	/
**BWA4C**	/	/	/	/	/	7.3^g^	/

Note: / = not determined.

a in vitro COX‐2 selectivity index, SI = *IC*_50_(COX‐1) / *IC*_50_(COX‐2).

b previously determined by us [53].

c could not be calculated.

d concluded from <50 % inhibition at 100 µM.

e p*IC*_50_ = 7.11 ± 0.05 (*n*  =  4).

f p*IC*_50_ = 8.11 ± 0.27 (*n*  =  2).

g determined at 0.3 µM (*n*  =  4).

### 2.3 5‐LO Inhibition

The inhibition of 5‐LO was determined by quantification of 5‐LO product formation in an intact cell polymorphonuclear leukocyte (PMNL) assay using *N*‐hydroxyacetamide derivative BWA4C ((*E*)‐*N*‐hydroxy‐*N*‐(3‐phenoxyphenyl)allyl)acetamide) as reference. BWA4C showed 7.3% residual activity at a concentration of 0.3 µM, which is in accordance with reported *IC*_50_ values (0.01–0.08 µM) [76, 77]. For both iodinated compounds **3** and **4**, an *IC*_50_ value toward 5‐LO of 0.62 and 0.54 µM, and hence in the sub‐micromolar range was observed (Table 1). A direct comparison to the nonhalogenated compounds was not possible because high variance of 5‐LO inhibition did not allow for *IC*_50_ determination of **1** and **2** [53]. However, the observed residual 5‐LO activity at inhibitor concentrations of 0.1 µM would suggest that iodination led to a slight decrease of 5‐LO inhibitory potency. In conclusion, **3** and **4** were found to be potent 5‐LO inhibitors and hence suitable for further evaluation.

### 2.4 Immobilized Artificial Membrane Chromatography

To probe potential interaction with cell membranes, compounds **1**–**4** were subjected to immobilized artificial membrane chromatography. Silica‐based columns with covalently bound phosphatidylcholine residues resemble biological membranes, creating a proportionality between the retention time and the Chromatographic Hydrophobicity Index (CHI_IAM_) of an analyte [78]. The higher this value the more pronounced the tendency of a drug molecule to interact with a biological membrane or micellar structures, as well as increased tendency to phospholipidosis for CHI_IAM_ above 50 [79, 80]. CHI_IAM_ values below 10 are typical for highly hydrophilic compounds. As comparison, CHI_IAM_ values for other NSAIDs have been reported between 20 and 40 [81]. The CHI_IAM_ obtained for **1**–**4** were between 45 and 50 (Table 2), suggesting that the carborane‐based compounds strongly interact with membranes. This in turn may impede membrane passage in favor of accumulation in phospholipid bilayers. Interestingly, for both iodinated compounds, slightly lower values compared to their nonhalogenated counterparts were observed.

**TABLE 2:** Log *D*_7.4_, CHI_IAM_ values, and plasma protein binding of **3** and **4** compared to precursors **1** and **2**.

Compound	Log *D*_7.4_	CHI_IAM_	Plasma protein binding in %
**1**	2.78^a^	48.1	/
**3**	1.99b,, c	45.8	95.7^c^
**2**	2.82^a^	49.3	/
**4**	1.66^c^	45.6	84.4b,, c

Note: / = not determined.

a previously determined by us using an HPLC method [53].

b *N* = 2, *n* = 3.

c determined using **[** ^**123**^ **I]3** and **[** ^**123**^ **I]4**, respectively.

### 2.5 Radioiodination

Initially, radioiodination of **1** was performed in aqueous solution using oxidants CAT, peracetic acid (AcOOH), and *N*‐chlorosuccinimide (NCS), respectively, at various temperatures (Table 3). Apart from radioiodination in the presence of AcOOH, both procedures gave moderate to good radiochemical conversions (RCC). Generally, AcOOH is employed in higher concentrations; 2 mM was used in consistency with CAT and NCS concentrations. Notably, when 2 mM AcOOH was generated in situ from AcOH and H_2_O_2_, the RCC was found to be significantly higher. The enhanced RCC may be explained by the presence of unreacted H_2_O_2_ in the reaction mixture. The radioiodinated main product was identical throughout the tested conditions and confirmed to be **[** ^**123**^ **I]3** by coinjection of reference compound **3** for radio‐high‐performance liquid chromatography (radio‐HPLC) analysis (Figure S 27, SI). Further, only negligible formation of radiolabeled byproducts (< 5%, HPLC) was observed in the tested reaction conditions. The reaction with CAT at room temperature (18–20°C) was chosen for further optimizing precursor demand and RCC. Precursor **1** and CAT were varied in a concentration range between 15.6 µM and 2 mM. High RCC values were found above a precursor concentration of 62.5 µM. For further manual radiosynthesis, 0.4 mM precursor and CAT were used to account for higher activity amounts and to maintain robust reaction conditions (further information Figure S 26, SI).

**TABLE 3:** Optimization of radioiodination of **1** with different oxidants (final concentrations given) and temperatures. Mixtures contained 2 mM **1** (final concentration), 5–10 MBq [^123^I]NaI and were allowed to react for 10 min at the given temperature. RCC was monitored by radio‐HPLC of the quenched reaction mixture diluted with 4 volume parts of 50% v/v CH_3_CN/H_2_O. Unless stated otherwise, *n *= 1.

		RCC in % (HPLC)			
Oxidant	Concentration of oxidant	rt	40°C	60°C	80°C
CAT	2 mM	95 (*n *= 3)	89	82	30 (*n *= 2)
AcOOH	In situ (approx. 2 mM)^a^	53 (*n *= 2)	/	/	/
	360 mM	32 (*n *= 2)	1	1	6
	36 mM	39	/	/	/
	2 mM	0	0	0	0
NCS	2 mM	96 (*n* = 2)	93	90	86

Note: / = not determined; rt = room temperature;

a 2 h prior to reaction, 20 µL of glacial acetic acid were mixed with 10 µL of 30% w/w hydrogen peroxide. Of this solution, 3 µL were transferred into a 50 µL reaction mixture. AcOOH concentration was estimated using equations of Zhao et al. [82].

**FIGURE 1:**
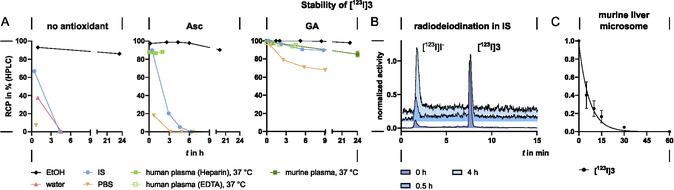
(A) Incubation of **[** ^**123**^ **I]3** in different media in the presence of antioxidants. All studies conducted at room temperature in the presence of 5% v/v EtOH, unless otherwise specified. (B) Radio‐HPLC of **[** ^**123**^ **I]3** after incubation in IS without added antioxidants. (C) Incubation of **[** ^**123**^ **I]3** with murine liver microsomes (*n* = 2, radio‐thin‐layer chromatography (radio‐TLC) Figure S32, SI).

Optimized conditions were adopted for manual radiosynthesis. The radiolabeled product could be separated from precursor **1** and byproducts by standard semipreparative HPLC protocols. Thereafter, solid‐phase extraction (SPE) using a reversed phase C_18_ cartridge was performed to remove the HPLC eluent and reformulate the radiotracer **[** ^**123**^ **I]3** in EtOH for further in vitro and in vivo investigations. We observed radiodeiodination of **[** ^**123**^ **I]3** over time in the collected HPLC fraction, which was avoided by immediate SPE after fraction collection. Notably, deiodination was observed in the final ethanolic solution leading to a radiochemical purity (RCP) of 86% after 23 h. Removal of EtOH under reduced pressure with a continuous flow of nitrogen at 70°C gave varying RCP (62%–100%). Further, storage in solutions of 5% v/v EtOH in isotonic saline (IS) or phosphate‐buffered saline(PBS) at pH 7.4 (PBS) showed even faster radiodeiodination, leading to an RCP of 67% at 25 min, and 7% at 40 min. After 4 h, **[** ^**123**^ **I]3** could no longer be detected in either solution (Figure 3B). To prevent radiodeiodination, we evaluated several antioxidants (Figure S 29, SI). Sodium ascorbate (Asc) or gentisic acid (GA) were finally added to the collected HPLC fraction, and the water used to wash the SPE (500 µL of a 1 mg/mL aqueous solution in each step) before elution with ethanol. Thus, the RCP of the final tracer **[** ^**123**^ **I]3** could be increased to > 97% using GA and to > 87% using Asc with increased shelf life of the radiotracer in EtOH (Figure 1A). However, stability of the radiotracer in 5% v/v EtOH in saline or PBS did not benefit from the antioxidants, so that the radiotracer was immediately diluted into buffer before further biological evaluation. Typical radiosyntheses starting from 95 to 690 MBq [^123^I]NaI furnished 59–310 MBq **[** ^**123**^ **I]3** in 39–67% RCY, synthesis time 70–90 min, A_m_ > 36 GBq/µmol, RC*P* > 97% (in the presence of GA) (further information Table S 1, SI).

The conditions established for the synthesis of **[** ^**123**^ **I]3** were applied to synthesize **[** ^**123**^ **I]4** and similarly allowed the synthesis and formulation of the second radiotracer in auspicious RCY and RCP (RCY 41–63%, *A*_m_ > 171 GBq/µmol, RC*P* > 93%, using GA as antioxidant, Table S 2, SI).

### 2.6 Physicochemical and Radiopharmacological In Vitro Evaluation

The shaking flask method was used to determine log *D*_7.4_ values of the radiotracers. The *para* isomer **[** ^**123**^ **I]4** exhibited a slightly lower log *D*_7.4_ value of 1.66 than **[** ^**123**^ **I]3** with log *D*_7.4_ of 1.99 (Table 2). Generally, the *para‐*carborane moiety is a more lipophilic building block than *meta‐*carborane [54]. This is also expressed in the slightly higher log *D*_7.4_ of **2** compared to **1** and in accordance with the longer retention times observed for **4** over **3** in all analytical (U)HPLC methods applied throughout (radio)synthesis. Log *D*_7.4_ of around two is suitable for membrane passage and would allow to reach COX‐2 or 5‐LO catalytic site while limiting unspecific off‐target binding due to high lipophilicity. The iodinated analogs **3** and **4** showed both slightly lower log *D*_7.4_ values compared to the noniodinated lead compounds **1** and **2** which might be explained by lower p*K*_a_ of the NH group induced by electron‐withdrawing nature (−I effect, weak +M‐effect) of the iodine lowering electron density at the thiophene. The different methods of lipophilicity determination may also account for the deviation.

Both radiotracers exhibited a high plasma protein binding as determined by ultrafiltration with Amicon spin columns (Table 2). High plasma protein binding may be preferable for avoiding fast metabolism and excretion of radiotracers [83]. Alternatively, as only the unbound fraction of radiotracer may accumulate into the target tissue, plasma protein binding may prolong circulation in the blood pool, and unspecific accumulation can be increased, both reducing signal‐to‐background ratio [84].

The stability of the radiotracers was investigated in various media, injectable solutions, and human and murine plasma. As discussed above, **[** ^**123**^ **I]3** showed radiodeiodination in EtOH, IS and PBS, which was more prominent in the aqueous solutions, which was also observed for **[** ^**123**^ **I]4** (Figures 1A and S 30, SI). Hence, Asc or GA were added to solutions of both radiotracers during the purification process, which improved the stability for **[** ^**123**^ **I]4**. The stabilization by GA was found to be superior to Asc. The formulations containing antioxidants merited investigation of plasma stability. Remarkably, the radiotracers showed higher stability than in the tested aqueous media. This observation was attributed to plasma protein binding properties of the compounds, as the bound compounds are likely somehow shielded from nonenzymatic deiodination in the medium. To test this hypothesis, a protein‐free ultrafiltrate of human plasma produced with Amicon spin columns (MWCO 10 kDa) was used to subject **[** ^**123**^ **I]3** to another stability test. Interestingly, the stability in the plasma filtrate was found to be superior to the stability in isotonic saline after 2.5 h (RCP 84% and 20%, respectively, Figure S 31, SI). The stability in plasma and the filtrate was found to be comparable. Therefore, we concluded that the presence of other redox‐systems in plasma, such as glutathione (GSH), is responsible for the higher stability, rather than plasma protein binding. Incubation in all media led to radiodeiodination without the detection of other radioiodinated degradation products (Figure 1B).

**[** ^**123**^ **I]3** was subjected to a stability test in murine liver microsomes. Samples were taken after 5, 10, 30, 60 min and were analyzed by radio‐TLC showing a rapid metabolism of the radiotracer within 30 min. With the exception of [^123^I]I^−^, no other metabolite could be detected. A biological half‐life of 4.8 min was calculated for **[** ^**123**^ **I]3** (Figure 1C). As described by Cavina et al., thiophenes are rather prone to in vivo deiodination [85]. It is likely that deiodination is the result of oxidative metabolism by cytochrome‐P450 (CYP450) enzymes. Murine liver microsomes reportedly also contain thyroxine 5^′^‐deiodinase‐1 (DIO1), which, physiologically, is responsible for deiodination of thyroid hormones T_4_, T_3_, and rT_3_ [86, 87]. In the absence of reducing agent NADPH, which acts as cofactor to CYP450, only 36% of the tracer remained intact after 60 min, indicating that there is an NADPH‐ and CYP450‐independent deiodination mechanism (nonenzymatic or due to ID‐1). Considering the rapid nonenzymatic deiodination in previous studies, the involvement of ID‐1 is possible, although without substantial effect.

### 2.7 Western Blot and Cell Uptake Studies

**FIGURE 2:**
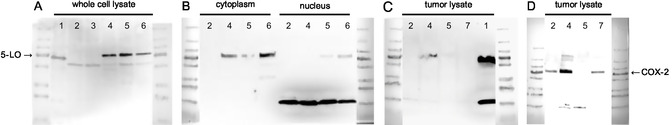
Western blot analysis with 5‐LO antibody (A–C) and COX‐2 antibody (D). (A) Whole cell lysates, NMRI^*nu*/*nu*^ lung tissue served as positive control for 5‐LO. (B) Fractionated cell lysates from cytoplasm and nucleus. (C) Tumor lysates after explantation from xenografted NMRI^*nu*/*nu*^ mice with NMRI^*nu*/*nu*^ lung tissue as positive control for 5‐LO. (D) Tumor lysates after explantation from xenografted NMRInu/nu mice with mouse pheochromocytoma cells as positive control for COX‐2. Samples are labeled as follows: (1) NMRI^*nu*/*nu*^ lung tissue, (2) U87 cells or tumor, (3) U87^COX−2KO^ cells, (4) HT‐29 cells or tumor, (5) MC cells or tumor, (6) M*Φ* cells, (7) mouse pheochromocytoma cells.

Cells for the studies were selected according to their COX‐2 and 5‐LO expression (Table 4). As reported, U87 and HT‐29 cells express high levels of COX‐2 [88, 89]. We recently reported on the CRISPR‐CAS mediated knockout of COX‐2 in U87 cells furnishing COX‐2 deficient U87^COX−2KO^ cells [90]. THP‐1 monocytes (MC) that do not express COX‐2 can be differentiated into resting M0 macrophages (M*Φ*) using phorbol‐12‐myristate‐13‐acetate (PMA). The differentiation is accompanied by an upregulation of COX‐2 expression [61, 90, 91, 92]. For determination of 5‐LO expression, cell lysates were analyzed by Western blot (Figure 2). In accordance with a previous study, 5‐LO was not detected by Western blot in both U87 cell lines [93]. In contrast, HT‐29, MC, and M*Φ* expressed 5‐LO [94, 95, 96]. The subcellular localization of 5‐LO in HT‐29 and THP‐1 was investigated using fractionated cell lysis combined with SDS‐PAGE and Western blot. 5‐LO was present in the cytoplasm of HT‐29, MC, and M*Φ*. As described previously, HT‐29 nuclear fraction did not contain 5‐LO protein in contrast to the leukocytes [94].

**TABLE 4:** COX‐2 and 5‐LO expression by cell type according to previous reports and/or present experimental data as indicated. Proteins were detected in lysates of cells and after explant of xenografted tumors by Western blot analysis.

Cell type	COX‐2		5‐LO (location)	
	Cell lysate	Tumor lysate	Cell lysate	Tumor lysate
U87	+[90]	+	—	—
U87^COX‐2KO^	−[90]	/	—	/
HT‐29	+[61]	+	+ (c)	+(/)
MC	−[61]	—	+ (c,n)	—
MΦ	+[61]	/	+ (c,n)	/

Note: + detected. − not detected. / not determined. c cytoplasmatic fraction. n nuclear fraction.

Cell uptake was investigated with radiotracers **[** ^**123**^ **I]3** or **[** ^**123**^ **I]4** alone (baseline) and in the presence of blocking agents, including **3**, **4**, ibuprofen, celecoxib, etoricoxib, zileuton, or EP6 (100 µM final concentration) [97, 98]. Notably, RP‐TLC analysis of cell supernatant after 180 min showed that the tracers were intact over the period of incubation within the medium (Figure S 31, SI). Celecoxib can be regarded as standard blocking agent in the development of COX‐2 radiotracers [56, 70]. However, in the case of COX‐2/5‐LO inhibitors, the 5‐LO inhibitory potential (*IC*_50_ 8 µM in ionophore‐activated PMNL) must be considered [99]. Therefore, etoricoxib was investigated as blocking agent targeting COX‐2 without concomitant 5‐LO inhibition (*IC*_50_ COX‐2 5.0 µM) [99, 100]. Ibuprofen was employed as COX‐1 selective inhibitor (*IC*_50_ of 12.70 and 368 µM for COX‐1 and COX‐2, respectively, determined using a comparable fluorescent assay as described before) [101]. *N*‐Hydroxyurea derivative zileuton acts via the complexation of the active site Fe^II^ cation of 5‐LO, thereby preventing enzyme activation by oxidation to ferric iron [102]. While there are three more classes of 5‐LO inhibitors, namely redox, competitive nonredox, and allosteric inhibitors, it is reasonable to assume that compounds **1**–**4** act via the same mechanism as zileuton, as proposed for the phenyl analog RWJ‐63 556 [52]. Nonetheless, by molecular docking approaches, other sulfonamide‐derived 5‐LO inhibitors have been ascribed allosteric modification of enzymatic activity [103, 104, 105]. Therefore, EP6, an allosteric inhibitor designed by Hieke et al. was also employed as a blocking agent hypothesized to bind to the C2 β‐barrel domain (and not comprising a sulfonamide) [97, 98].

**FIGURE 3:**
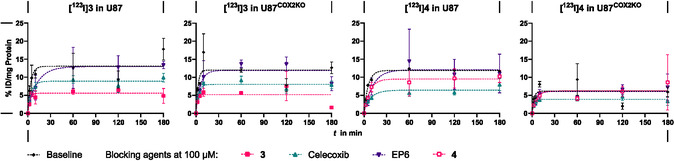
In vitro study of cellular uptake of **[** ^**123**^ **I]3** (0.14 MBq/mL, > 62.3 MBq/nmol) and **[** ^**123**^ **I]4** (0.16 MBq/mL, >171 MBq/nmol) in U87 and U87^COX−2KO^ cells over 180 min. Results are given as mean ± SD, represented as % initial dose (ID) per mg protein. Cells were preincubated with 100 µM of the corresponding blocking substance for 30 min before addition of the radiotracers. *n* = 3.

**[** ^**123**^ **I]3** and **[** ^**123**^ **I]4** were taken up showing saturation binding by both U87 and U87^COX−2KO^. The plateau was reached within 30 min at a binding capacity of about 5–12% ID/mg protein (Figures 3 and S 34–35, SI). Comparing the baseline uptake, there was no significant difference between **[** ^**123**^ **I]3** and **[** ^**123**^ **I]4**, which was as expected regarding their overall similar properties as regioisomers. Between U87 and U87^COX−2KO^, no differences in the height of plateau nor time to achieve saturation was observed for both tracers, suggesting that COX‐2 is not involved in cellular uptake (13% ID/mg protein and 12% ID/mg protein after 60 min for **[** ^**123**^ **I]3**). Celecoxib led to a decreased cell binding for both radiolabeled compounds in COX‐2 expressing and COX‐2KO cells. The uptake of **[** ^**123**^ **I]3** was blocked by compound **3** in both cell lines, while the nonradioactive reference **4** blocked uptake of **[** ^**123**^ **I]4** only in U87 cells. In contrast, no blocking was observed in the presence of etoricoxib or any of the other blocking substances employed. Since ibuprofen did not elicit a decrease in uptake, a contribution of COX‐1 is also unlikely. The observations in cells incubated with COX inhibitors further support the conclusion of COX‐2 independent uptake and suggest a shared path of entry for the radiotracers and celecoxib.

**FIGURE 4:**
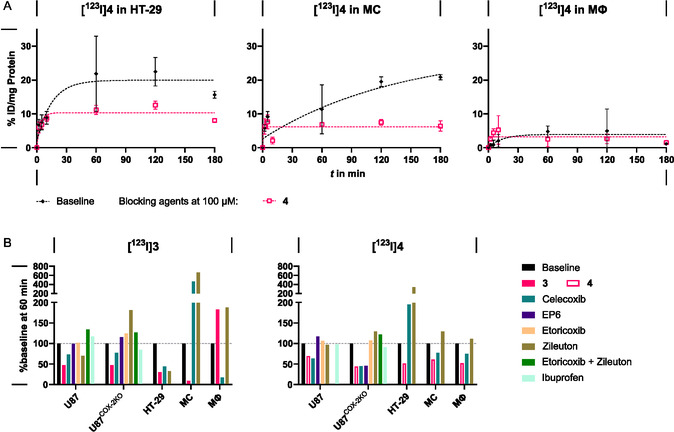
(A) In vitro study of cellular uptake of **[** ^**123**^ **I]4** in HT‐29, MC and MΦ cells over 180 min (0.25 MBq/mL with >352 MBq/nmol for HT‐29 and MΦ; 0.90 MBq/mL, >352 MBq/nmol for MC). Results are given as mean ± SD, represented as % ID per mg protein. Cells were preincubated with 100 µM of the corresponding blocking substance for 30 min before addition of the radiotracer. *n* = 3. (B) Blocking of radiotracer uptake (**[** ^**123**^ **I]3** and **[** ^**123**^ **I]4**, respectively) in U87, U87^COX−2KO^, HT‐29, MC and MΦ in the presence of blocking agents after 60 min of radiotracer incubation. Data are normalized to baseline uptake of radiotracer in the respective cell line. Cells were preincubated with 100 µM of the corresponding blocking substance for 30 min before addition of the radiotracers. *n* = 3.

To investigate a possible involvement of 5‐LO alone or in combination with COX‐2 in cellular uptake studies, HT‐29 cells and MC were used. HT‐29 cells express both target enzymes, while MC in unstimulated form express only 5‐LO but can be triggered to differentiate to resting macrophages, which then express COX‐2. Blocking agents ibuprofen and etoricoxib were not used as both COX isoforms appear not to be involved in cell uptake. In fact, HT‐29 exhibited an increased uptake of **[** ^**123**^ **I]3** and **[** ^**123**^ **I]4** (49% and 22% ID/mg protein after 60 min, respectively) compared to U87 (Figures 4A and S 34–35, SI). Both tracers reached saturation within 60 min. The *meta* isomer showed a lower uptake in the presence of celecoxib, zileuton, or **3**. The increased and by 5‐LO inhibitors partially blockable uptake of **[** ^**123**^ **I]3** would support that 5‐LO is involved in cellular uptake and retention in HT‐29 cells. For **[** ^**123**^ **I]4**, only the nonradioactive reference elicited blocking of uptake, while neither celecoxib nor zileuton did so. Hence, **[** ^**123**^ **I]4** uptake is likely independent of 5‐LO in HT‐29.

In contrast, MC exhibited higher uptake of **[** ^**123**^ **I]4** (10–20% ID/mg protein) compared to M*Φ* at baseline (5% ID/mg protein). Saturation was reached for both cell lines. Compound **4** and celecoxib blocked uptake of **[** ^**123**^ **I]4** in MC. None of the blocking agents elicited a decrease in uptake in M*Φ*. Uptake behavior of **[** ^**123**^ **I]3** in MC remained inconclusive, while in M*Φ* a similar uptake to **[** ^**123**^ **I]4** was observed. In summary, while for HT‐29 involvement of 5LO in cellular uptake of **[** ^**123**^ **I]3** is indicated, uptake behavior in M*Φ* with higher expression of 5‐LO compared to the MC cells contradicts sole 5‐LO specific uptake in these cells. In all cell lines, a nonblockable uptake of the tracers **[** ^**123**^ **I]3** and **[** ^**123**^ **I]4** was observed and the presence of off‐targets or nonspecific accumulation into the membrane, as indicated by high CHI_IAM_ values need to be considered as contributing factors. A contribution of COXs in cellular accumulation can practically be excluded, which is in accordance with only moderate COX inhibition potency of **3** and **4**.

### 2.8 In Vivo Studies

**FIGURE 5:**
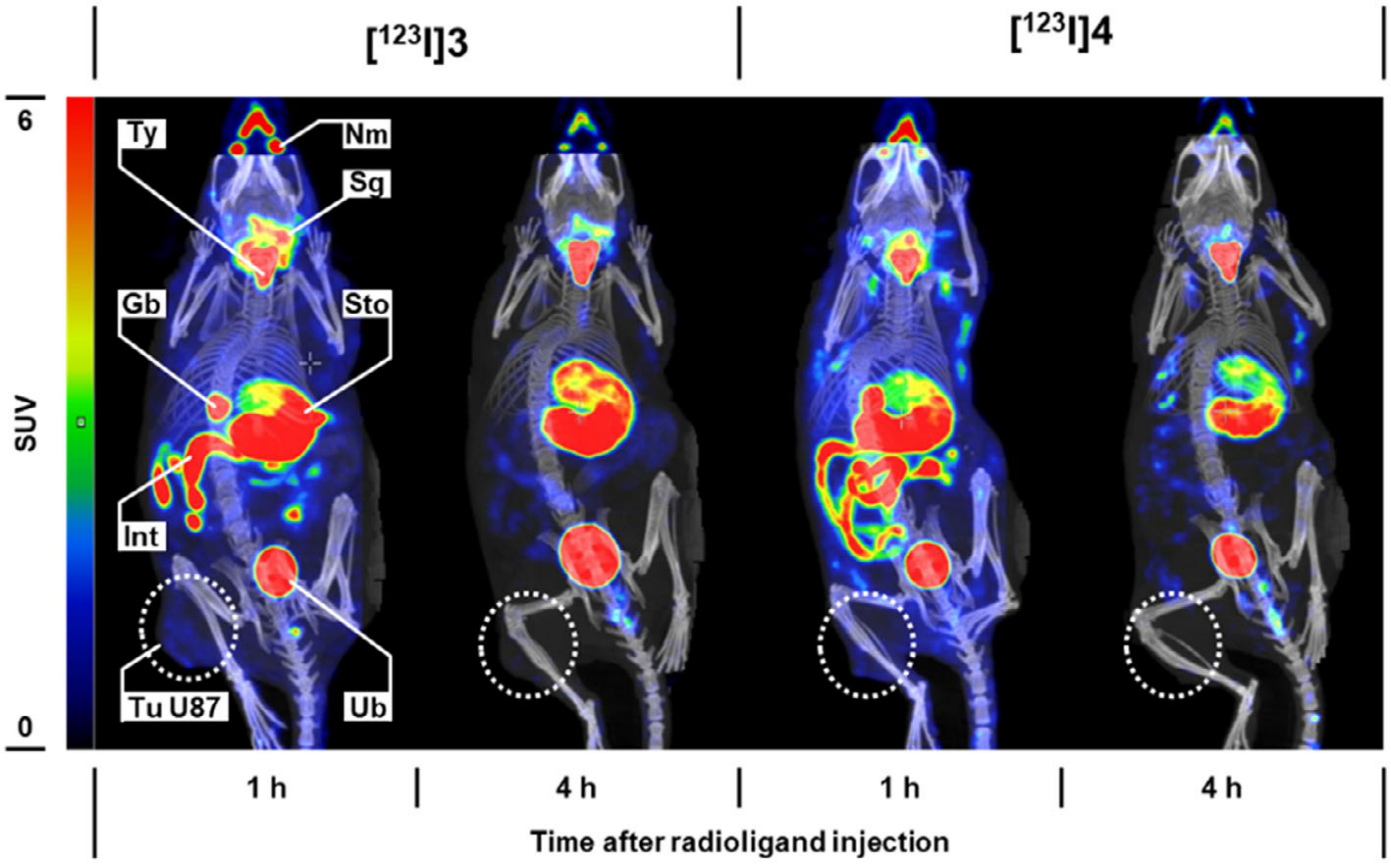
Distribution of **[** ^**123**^ **I]3** and **[** ^**123**^ **I]4** in mice visualized using quantitative SPECT imaging. Maximum‐intensity projections of U87 tumor‐bearing mice at indicated time points after intravenous injection of the radiolabeled compounds (n.c.a), each administered at an initial dose between 11.8 and 26.4 MBq in presence of sodium ascorbate as antioxidant. (Gb) gall bladder, (Int) intestine, (Nm) nasal mucosa, (Sg) salivary glands – sublingual and submandibular, (Sto) stomach, (Tu) tumor, (Ty) thyroid, (Ub) urinary bladder, (SUV) standardized uptake value.

To corroborate the observations made during in vitro analysis, the distribution of the radiolabeled compounds was investigated in mice bearing subcutaneous U87, HT‐29, and MC tumor xenografts by SPECT imaging. Since M*Φ* de‐differentiate when their stimulus (PMA) is no longer present, they do not form solid tumors [106, 107] and were therefore not included in the biodistribution studies. U87^COX−2KO^ was also excluded considering its similar behavior to U87 in vitro. In SPECT images, both **[** ^**123**^ **I]3** and **[** ^**123**^ **I]4** showed predominant uptake in the stomach wall, thyroid gland, and salivary glands (representative images of U87 tumor‐bearing mice: Figure 5), pointing toward rapid radiodeiodination and uptake of [^123^I]I^−^ by these Na^+^/I^−^ symporter (NIS)‐positive tissues [108, 109, 110]. Up to 30 % of the initially administered dose were eliminated within 1 h via urine (Table 5). Urine was analyzed shortly after SPECT imaging via radio‐TLC and revealed that no intact radiolabeled compound was eliminated via the renal pathway, but that the entire activity could be assigned to [^123^I]I^−^ (Figure S 38, SI). These observations are in accordance with the results of the MLM assay, which suggested a rapid radiodeiodination by hepatic enzymes leading to clearance of [^123^I]I^−^ by the kidneys and typical distribution pattern into NIS‐positive tissues. The fact that no accumulation of activity was observed in the liver further supports this conclusion. Activity in the gall bladder and gastrointestinal tract can be ascribed to [^123^I]I^−^ excretion via the bile duct and/or the gastric mucus.

**TABLE 5:** SPECT image‐extracted uptake values (% ID) characterizing the retention and elimination of **[** ^**123**^ **I** **]** **3** and **[** ^**123**^ **I** **]** **4** in mice.

	[^123^I]3		[^123^I]4	
	1 h (*n* = 6)	4 h (*n* = 4)	1 h (*n* = 8)	4 h (*n* = 3)
Retention total	65.2 ± 4.01	36.5 ± 4.29	63.9 ± 1.60	36.7 ± 6.33
NIS‐positive tissues^a^	22.8 ± 1.87	24.3 ± 4.23	20.4 ± 0.80	22.0 ± 3.51
Excretion total	34.8 ± 4.01	63.5 ± 4.29	36.1 ± 1.60	63.3 ± 6.33
renal fraction^b^	27.8 ± 4.18	n.d.	30.1 ± 1.39	n.d.
hepatobiliary fraction^c^	7.17 ± 1.68	n.d.	6.13 ± 0.55	n.d.

Note: Data presented as percent of the initially administered dose (% ID); means ± standard error; *n*indicates the number of animals investigated;

a major Na^+^/I symporter (NIS)‐positive tissues: thyroid + salivary glands + stomach;

b urinary bladder + (100% – remainder);

c gall bladder + intestine, assuming that the entire hepatobiliary fraction is still detectable inside the animal after 1 h

Both **[** ^**123**^ **I]3** and **[** ^**123**^ **I]4** did not show uptake in U87, HT‐29, or MC tumor xenografts, irrespective of the antioxidant used in the formulations (see Figure S 36, SI for complete panel of SPECT images). Of note, initial and preliminary results indicated uptake of **[** ^**123**^ **I]4** (with Asc) in the peripheral region of a U87 tumor (Figure S 37, SI), but not when the formulation contained GA as antioxidant [111]. Therefore, GA was tested for its COX inhibitory potency (Table 1). In line with previous reports [112], we found that the salicylic acid metabolite GA exhibits *IC*_50_ values within the micromolar range (73 and 2.4 µM for COX‐1 and COX‐2, respectively) and may be considered a competitive agent in the radiotracer formulation. Nevertheless, cells pretreated with 100 µM GA did not show any decrease in uptake of **[** ^**123**^ **I]3** or **[** ^**123**^ **I]4** (Figures S 34–35, SI). To exclude any possible influence of GA on the distribution of the radiolabeled compounds, all further studies were conducted with Asc in the formulations. However, imaging data from the completed series of independent in vivo experiments did not confirm the preliminary evidence for tumor‐specific uptake of **[** ^**123**^ **I]4**. Furthermore, both **[** ^**123**^ **I]3** and **[** ^**123**^ **I]4** also did not accumulate in the lung, known as a 5‐LO‐positive tissue. Tumor xenografts were explanted and analyzed by Western blot for target enzyme expression (Figure 2C,D; Table 4). U87 and HT‐29 exhibited the same expression pattern as expected from the cell culture. MC did not express COX‐2, as expected, but 5‐LO was also not detectable in the tumor lysate.

## 3 Material and Methods

Chemicals and solvents were purchased from Sigma–Aldrich Laborchemikalien GmbH, Merck KGaA, abcr GmbH, BLD Pharmatech GmbH, Fisher Scientific GmbH, Carl Roth GmbH & Co. KG, and were used without further purification. Deuterated solvents were purchased from Deutero GmbH. Dry solvents were purchased from Sigma–Aldrich Laborchemikalien GmbH in Sure/Seal bottles. HPLC‐grade water was purified from deionized water using a Mill‐Q Integral 5 system by Merck KGaA.

NMR spectra were recorded on the following spectrometers using the given conditions: Bruker Avance DRX 400 Spectrometer (26°C, ^1^H NMR 400.16 MHz, ^11^B NMR 128.38 MHz, ^13^C NMR 100.63 MHz) and Agilent DD2‐400 MHz Agilent Technologies 400 MR spectrometer consisting of 400/54 premium compact magnet, 400 MR console, and 400 MHz OneNMRProbe PT probe (25°C, ^1^H NMR 399.95 MHz, ^11^B NMR 128.32 MHz, ^13^C NMR 100.58 MHz). Chemical shifts (δ) are reported in ppm. ^1^H NMR spectra were either referenced to Si(CH_3_)_4_ or to the solvent residual signal as internal standard for ^1^H and ^13^C: CDCl_3_ (δ_H_ = 7.26 ppm; δ_C_ = 77.2 ppm), or (CD_3_)_2_SO (δ_H_ = 2.50 ppm; δ_C_ = 39.5 ppm) [113]. ^11^B chemical shifts were calculated according to the Ξ scale [114]. The ^11^B‐NMR experiments were carried out with Deutero quartz NMR tubes. Spectra were processed using MestreNova (version 15.0.1−35756). Analyses followed first order, and the following abbreviations were used throughout: s = singlet, d = doublet. Coupling constants (*J*) are given in Hz and refer to B, H‐couplings.

High resolution mass spectra (HR‐MS) were obtained as ESI mass spectra using a Bruker Daltonics Apex II FT‐ICR spectrometer (HR‐MS1) or a Q‐TOF MS: Agilent 1260 Infinity II HPLC (Santa Clara, California, USA; pump G7104C, autosampler G7129C, column oven G7116A, DAD detector G7117C) coupled to gamma detector Gabi Star (Elysia‐raytest GmbH, Straubenhardt, Germany) followed by accurate mass Revident Q‐TOF LC/Q‐TOF G6575A (HR‐MS2). Unless otherwise stated, the measurements were performed in bypass mode using an eluent consisting of (A): CH_3_CN and (B): 0.1 % formic acid in H_2_O; flow rate 0.2 mL/min). A reference mass solution containing hexakis(1H, 1H,3H‐tetrafluoropropoxy)phosphazene, and purine was continuously coinjected via dual AJS ESI source. The system was operated using Agilent Masshunter Workstation 3.6 – LC/MS data aquisition software (Version 12.0) and data evaluation was performed using Agilent Masshunter Workstation 3.6 Qualitative Analysis software (Version 12.0 Update 1).

TLC was performed on Merck TLC Silica gel 60 F_254_ aluminum sheets (normal phase (NP), Merck KGaA, Darmstadt, Germany) or precoated TLC‐sheets ALUGRAM RP‐18W/UV_254_ (reversed phase (RP), Macherey‐Nagel GmbH & Co. KG, Düren, Germany) with visualization under UV (254 nm). Carborane‐containing substances were stained and identified with a 5 % solution of palladium(II) chloride in methanol. Radio‐TLC was performed as described for TLC and visualized using CR35 Bio (Elysia‐raytest GmbH, Straubenhardt, Germany) or Amersham Typhoon 5 (Cytiva Germany GmbH, Dreieich, Germany). Chromatograms were analyzed using advanced image data analyzer (AIDA) software (v5.1 SP4, Raytest, Straubenhardt, Germany).

Preparative HPLC was performed on Shimadzu LC‐20A Prominence HPLC consisting of a degasser unit DGU‐20A5R, two separate pumping units LC‐A20R, sample manager SIL‐20ACHT, column oven CTO‐20AC, PDA‐detector SPD‐M20A, communication‐bus module CBM‐20A, fraction collector FRC‐10A, and Phenomenex Jupiter Proteo C18 column (250 mm × 21.2 mm, 4 µm, 90 Å). A binary linear gradient system of 0.1 % CF_3_COOH/H_2_O (A) and 0.1 % CF_3_COOH/CH_3_CN (B) at a flow rate of 10 mL/min and column temperature of 50°C served as the eluent. LabSolutions Software V. 5.92 was used for data processing. Gradient system 1 (%B): *t*_0 min_ 70 – *t*_5 min_ 70 – *t*_25 min_ 95 – *t*_31 min_ 95 – *t*_32 min_ 70 – *t*_42 min_ 70.

UPLC−DAD‐MS was performed on a Waters ACQUITY UPLC I class system including an ACQUITY UPLC PDA e *λ* detector coupled to a Xevo TQ‐S mass spectrometer and equipped with an ACQUITY Premier Peptide BEH C18 column (100 mm × 2.1 mm, 1.7 μm, 300 Å) along with an ACQUITY Premier Peptide BEH C18 VanGuard Pre‐column (5 mm × 2.1 mm, 1.7 μm, 300 Å). A binary linear gradient system of 0.1% CH_3_COOH/H_2_O (A) and 0.1% CH_3_COOH in CH_3_CN/CH_3_OH (1:1, v/v, B) at a flow rate of 0.4 mL/min and a column temperature of 50°C served as the eluent. MassLynx (v4.2 SCN986) was used for data processing. Gradient system 2 (%B): *t*_0 min_ 45 – *t*_0.5 min_ 45 – *t*_5.5 min_ 95 – *t*_7 min_ 95 – *t*_8 min_ 45 – *t*_8.5 min_ 45. ESI^−^ mode was used.

HPLC‐DAD was performed on Shimadzu Nexera X2 UHPLC system (Kyoto, Japan), equipped with degassers DGU‐20A3R and DGU‐20A5R, pump LC‐30AD, autosampler SIL‐30AC, column oven CTO‐20AC with two column switching valves FCV‐14AH, diode array detector SPD‐M30A, gamma detector GABI Star (Elysia‐raytest GmbH, Straubenhardt, Germany), communication bus module CBM‐20A, and a Kinetex C18 column from Phenomenex (250 mm × 4.6 mm, 5 µm, 100 Å). A binary gradient system of 0.1 % v/v CF_3_COOH/H_2_O (A) and CH_3_CN (B) at a flow rate of 1 mL/min and column temperature of 40°C served as the eluent. Gradient system 3 (%B): *t*_0 min_ 45 – *t*_10 min_ 45 – *t*_11 min_ 95 – *t*_16 min_ 95 – *t*_17 min_ 45 – *t*_25 min_ 45.

Semi‐preparative radio‐HPLC purification was performed on Jasco HPLC system equipped with LC‐NetII/ADC interface, PU‐2080 Plus pump, a Ternary Gradient Unit LG‐980−02, degasser DG‐980−50, a UV‐2075 Plus detector (Jasco Corporation, Tokyo, Japan), a gamma detector GABI (Elysia‐Raytest GmbH, Straubenhardt, Germany) and a Phenomenex Onyx C18 Monolithic Semi‐Prep column (100 × 10 mm, 130 Å). The purification was carried out using an isocratic system of 50% v/v CH_3_CN/H_2_O over 33 min at a flow rate of 4 mL/min at room temperature (18–20°C) (system 4). After each purification, the column was flushed with 95 % v/v CH_3_CN/H_2_O for 5–7 min. Data was processed through Jasco ChromNAV Software version 2.02.05.

Analytical (radio‐) HPLC was performed on Agilent 1200 Series (Agilent Technologies, Santa Clara, CA; system 5) consisting of interface 35900E, quaternary pump G1311A, degasser G1322A, autosampler G1329A, thermostatted column compartment G1316A, and diode array detector G1315D, equipped with a gamma detector GABI (Elysia‐raytest GmbH, Straubenhardt, Germany) and Purospher RP‐18 endcapped (5 µm) LiChroCART 125–3 column (125 × 3 mm, 5 μm, 120 Å). A binary gradient sytem of 0.1% v/v CF_3_COOH in H_2_O (A) and CH_3_CN (B) at 40°C was used. Data was processed using OpenLAB CDS ChemStation Edition version C.01.07 SR1. System 5, gradient 1 (%B): *t*_0 min_ 61 – *t*_10 min_ 61 – *t*_11 min_ 95 – *t*_16 min_ 95 – *t*_17 min_ 61 – *t*_22 min_ 61, flow rate 1 ml/min; system 5 gradient 2 (%B): *t*_0 min_ 5 – *t*_3 min_ 5 – *t*_28 min_ 95 – *t*_29 min_ 95 – *t*_35 min_ 5 – *t*_40 min_ 5, flow rate 0.75 ml/min; system 5 gradient 3 (%B) *t*_0 min_ 45 – *t*_3 min_ 45 – *t*_28 min_ 95 – *t*_29 min_ 95 – *t*_35 min_ 45 – *t*_40 min_ 45, flow rate 0.75 ml/min.

Chemical formulas were drawn and m/z calculated using ChemDraw Professional 19.1.1.21.

The no‐carrier added [^123^I]NaI was produced in‐house using a TR‐Flex cyclotron (Advanced Cyclotron Systems Inc., ACSI, Canada) and the gas target KIPROS 200 from ZAG Zyklotron AG (Eggenstein‐Leopoldshafen, Germany) by bombardment of highly enriched [^124^Xe]xenon gas with 30 MeV protons via, among others, the nuclear reaction ^124^Xe(p, pn)^123^Xe → ^123^I. Concentration of crude [^123^I]iodide and formulation in 0.02 M aqueous NaOH was performed by ROTOP Radiopharmacy GmbH (Dresden, Germany). Aliquots containing [^123^I]iodide in an activity concentration of 20–50 MBq/µL were used for further experiments and diluted accordingly with 0.02 M NaOH.

### 3.1 Chemical Synthesis

**3** (*N*‐[5‐(*closo*−1,7‐dicarbadodecaborane(12)‐1‐ylmethyl)‐3‐iodothiophen‐2‐yl]methanesulfonamide): At room temperature (18–20°C), 2.75 mL of 4 mM aqueous chloramine‐T solution (2.6 mg, 0.011 mmol, 1.1 eq) were added dropwise to a mixture of 115 µL 100 mM NaI solution in 0.02 M aqueous NaOH and **1** (3.5 mg, 0.010 mmol, 1.0 eq, in beforehand dissolved in 1.1 mL DMSO) in a 10 mL round bottom flask. Progress of the reaction was monitored by system 2. After 75 min, the reaction was quenched with 2.0 mL 100 mM aqueous Na_2_S_2_O_5_ solution (0.20 mmol, 20 eq). The suspension was filtered via fritted glass funnel and washed with 5 mL of water twice. For transfer, the solid was dissolved in EtOH which was evaporated under reduced pressure. For purification with system 1, the crude product was dissolved in 70 % v/v CH_3_CN/H_2_O. The product fractions were combined, diluted with the 2.5‐fold volume of water and lyophilized. This yielded **3** as a white solid (2.75 mg, 0.006 mmol, 60 %). RP‐TLC (60 % v/v CH_3_CN/H_2_O) *R*_f_ = 0.41, ^1^H NMR (400 MHz, CDCl_3_): δ = 6.69 (s, 1 H, C*H*_thiophen_), 6.35 (s, 1 H, N*H*), 3.30 (s, 2 H, C*H*_2_), 3.08 (s, 3 H, C*H*_3_), 2.93 (s, 1 H, C*H*_carboran_), 2.79–1.23 (br, 10 H, B*H*). ^11^B{^1^H} NMR (128 MHz, CDCl_3_) δ = −3.9 (s, 2 B, *B*H), −9.5 (br, 1 B, *B*H), −10.5 (s, 2 B, *B*H), −10.9 (shoulder, 1 B, *B*H), −13.3 (s, 2 B, *B*H), −15.4 (s, 2 B, *B*H). ^11^B NMR (128 MHz, CDCl_3_) *δ* = −3.9 (d, *J* = 161 Hz, 2 B, *B*H), −9.5 (d, *J* = 123 Hz, 1 B, *B*H), −10.5 (d, *J* = 140 Hz, 2 B, *B*H), −10.8 (d, *J* = 202 Hz, shoulder, 1 B, *B*H), −13.3 (d, *J* = 165 Hz, 2 B, *B*H), −15.4 (d, *J* = 181 Hz, 2 B, *B*H). ^13^C{^1^H} NMR (100 MHz, CDCl_3_): *δ* = 138.4 (thiophen‐*C*_5_), 136.2 (thiophen‐*C*_3_), 132.6 (thiophen‐*C*_4_), 74.8 (carborane‐*C*_1_), 55.4 (carborane‐*C*_7_), 40.8 (*C*H_3_), 37.1 (*C*H_2_). HR‐ESI‐MS (negative mode, CH_3_CN) m/z [M−H]^−^ calculated for C_8_H_17_B_10_INO_2_S_2_^−^: 458.0754, found: 458.0747 (HR‐MS1), the observed isotopic pattern agreed with the calculated one. HPLC *t*_R_ = 23.3 min purity 97 % (254 nm, system 5, gradient 2).

**4** (*N*‐[5‐(*closo*−1,12‐dicarbadodecaborane(12)‐1‐ylmethyl)‐3‐iodothiophen‐2‐yl]methanesulfonamide): At room temperature (18–20°C), 3.30 mL of 4 mM aqueous chloramine‐T solution (3.0 mg, 0.013 mmol, 1.1 eq) were added dropwise to a mixture of 132 µL 100 mM NaI solution in 0.02 M aqueous NaOH and **2** (4.0 mg, 0.012 mmol, 1.0 eq, in beforehand dissolved in 1.5 mL DMSO) in a 10 mL round‐bottom flask. Progress of the reaction was monitored by system 2. After 60 min, the reaction was quenched with 2.4 mL of 100 mM aqueous Na_2_S_2_O_5_ solution (0.24 mmol, 20 eq) and diluted to 20 mL with water. The mixture was extracted with 15 mL EtOAc twice. The organic phases were combined, washed with brine and evaporated under reduced pressure. For purification with system 1, the crude product was dissolved in 70 % v/v CH_3_CN/H_2_O. The product fractions were combined, diluted with the 2.5‐fold volume of water and lyophilized. This yielded **4** as a pale‐yellow solid (0.76 mg, 0.002 mmol, 14 %). RP‐TLC (50 % v/v CH_3_CN/H_2_O) *R*_f_ = 0.30, ^1^H NMR (400 MHz, (CD_3_)_2_SO): δ = 9.85 (s, 1 H, N*H*), 6.71 (s, 1 H, C*H*_thiophen_), 3.70 (s, 1 H, C*H*_carboran_), 3.11 (s, 2 H, C*H*_2_), 3.01 (s, 3 H, C*H*_3_), 2.67–1.24 (br, 10 H, B*H*). ^11^B{^1^H} NMR (128 MHz, CDCl_3_) δ = −12.7 (s, 5 B, *B*H), −14.9 (s, 5 B, *B*H). ^11^B NMR (128 MHz, CDCl_3_) δ = −12.8 (d, *J* = 164 Hz, 5 B, *B*H), −14.9 (d, *J* = 183 Hz, 5 B, *B*H). ^13^C{^1^H} NMR (100 MHz, (CD_3_)_2_SO; supplemented with signals from 2D spectra as indicated): δ = 138.5 (from HMBC, thiophen‐*C*_5_), 136.6 (from HMBC, thiophen‐*C*_3_), 133.4 (thiophen‐*C*_4_), 81.8 (from HMBC, carborane‐*C*_1_), 59.4 (from HSQC, carborane‐*C*_12_), 40.7 (*C*H_3_), 37.4 (*C*H_2_). HR‐ESI‐MS (negative mode) m/z [M−H]^−^ calculated for C_8_H_17_B_10_INO_2_S_2_^−^: 458.0754 found: 458.0755 (HR‐MS2), the observed isotopic pattern agreed with the calculated one. HPLC *t*_R_ = 4.3 min, purity > 99 % (254 nm, system 5, gradient 3).

### 3.2 COX Inhibition Assay

The COX inhibition activity against ovine COX‐1 and human recombinant COX‐2 was determined using the COX Fluorescent Inhibitor Screening Assay Kit (Cayman Chemical Company, Ann Arbor, MI, USA) according to the manufacturer's instructions as reported [115]. The compounds were screened at a concentration of 100 µM in duplicate. For determination of the *IC*_50_ values, compounds were assayed in a concentration range from 0.32 to 320 µM in duplicate. *IC*_50_ values were determined with GraphPad Prism 10 (v10.4.2) by fitting to the equation y = A_2_ + (A_1_ − A_2_) / (1 + (x / x_0_)^p^) and are given as absolute *IC*_50_ values. COX‐2 selective inhibitor celecoxib and COX‐1 selective inhibitor SC‐560 served as reference compounds (Table 1).

### 3.3 5‐LO Inhibition Assay

The assay was performed with freshly isolated PMNL from human buffy coats. 5 × 10^6^ cells were resuspended in 1 mL PBS containing 1 mg/mL glucose and 1 mM CaCl_2_. Cells were preincubated with inhibitor for 15 min at 37°C before starting the reaction by the addition of 2.5 µM ionophore and 20 µM arachidonic acid. After 10 min, the reaction was stopped with 1 mL ice‐cold methanol. Solid phase extraction (SPE) and UHPLC analysis was performed as described before [116]. *IC*_50_ values were determined with GraphPad Prism (v7.05) using a nonlinear regression with variable slope. *n* = 4.

### 3.4 Radiosynthesis of [^123^I]3 and [^123^I]4

5 µL of precursor **1** and **2**, respectively, in a 10 mM stock solution in DMSO was mixed with 57.5 µL water and 50 µL 0.02 M aqueous H_3_PO_4_. 12.5 µL of 4 mM aqueous CAT solution was added, followed by 50–800 MBq [^123^I]NaI in 50 µL 0.02 M aqueous NaOH. After 10 min, the reaction was quenched by adding 10 µL of 100 mM aqueous Na_2_S_2_O_5_. The crude product was diluted with 1.6 mL 35 % v/v CH_3_CN/H_2_O and purified using system 4. In the fraction collector, 0.5 mL aqueous antioxidant solution (Asc or GA, 1 mg/mL) was placed in beforehand in the collection falcon tube. The collected fraction was diluted with 20 mL H_2_O and transferred to a CHROMAFIX C_18_ ec (S) SPE cartridge (Macherey‐Nagel GmbH & Co. KG, Düren, Germany). The cartridge was rinsed with 5 mL aqueous antioxidant solution (Asc or GA, 0.1 mg/mL) before elution with 1 mL EtOH. The obtained solution was concentrated at 70°C under reduced pressure and a gentle stream of nitrogen gas. In the SI, an overview of activity yield, RCY, RCP, and *A*_m_ is provided (Tables S 1–2, SI). Activity yield and RCY were determined using an ISOMED 2000 activimeter (MED Nuklear‐Medizintechnik Dresden GmbH, Dresden, Germany), RCP and *A*_m_ using radio‐HPLC (system 5 gradient 1).

### 3.5 Log *D*_7.4_ Determination

Log *D*_7.4_ was determined with the shaking flask method. The tracer solution in EtOH as obtained after SPE was evaporated to dryness under reduced pressure and a gentle flow of nitrogen gas at 70°C. The residue was taken up in 100 µL of organic phase (*n*‐octanol saturated with PBS pH 7.4). Aliquots of 25 µL were diluted to 900 µL and added to an equal volume of aqueous phase (PBS pH 7.4 saturated with *n*‐octanol). The mixture was shaken vigorously for 30 s and centrifuged at 16,100 *g* for 5 min at 20°C. 600 µL of the organic phase were transferred to another vial containing the same amount of aqueous phase. The mixture was shaken and centrifuged as before, and the activity of aliquots of both phases was measured using a well‐type counter (ISOMED 2100, NUVIA Instruments GmbH, Dresden, Germany). Log *D*_7.4_ was determined from the decay‐corrected activities: $\log\;D_{7.4}\;=\;\log\;(A_{n‐oct}\;/\;A_{PBS})$.

### 3.6 Immobilized Artificial Membrane Chromatography

CHI_IAM_ values were experimentally determined using an HPLC method developed by Valko et al. [117] and previously described by us [62]. In brief, substances were injected on an Agilent 1200 system, equipped with an IAM PC DD2 column (100 × 4.6 mm, 10 µM, Regis Technologies), using a linear gradient of 50 mM ammonium acetate pH 7.4 (A) and CH_3_CN (B) as eluent: (%B) *t*_0 min_ 0 – *t*_9 min_ 100 – *t*_9.5 min_ 100 – *t*_10.5 min_ 0, flow rate 1 mL/min. Acetanilide, acetophenone, anisole, benzoic acid, 1,4‐dinitrobenzene, propiophenone, valerophenone, and octanophenone served as references.

### 3.7 Plasma Separation from Whole Blood

For human plasma, venous blood (4.5 mL) from one healthy, male volunteer who was not fasting or on any medication was collected into Vacuette LH Lithium Heparin plasma separator tubes (Greiner Bio‐One GmbH, Frickenhausen, Germany). For mouse plasma, arterial blood (>1 mL) from an NMRI^*nu*/*nu*^ mouse was obtained by heart punctuation. Blood samples were dispensed into lithium heparin (Heparin‐Sodium LEO 25.000 I.U./5 mL) flushed 1.5 mL Eppendorf tubes. The tubes were allowed to stand on ice for 30 min protected from light followed by centrifugation at 2000 *g* for 15 min at 20°C (human) or 2000 *g* for 10 min at 4°C (mouse). Samples were visually checked for hemolysis and interference; the plasma layer was frozen in liquid nitrogen and lyophilized. The resulting powder was stored at 4°C (protected from light) and reconstituted by adding the according amount of water prior to use.

### 3.8 Ultrafiltration Assay

An ultrafiltration assay was used to determine the binding of the iodine‐123 labeled compounds to plasma proteins. Amicon Ultra‐0.5 centrifugal filters (MWCO 10 kDa, Merck KGaA, Darmstadt, Germany) were used to separate the free fraction of the radioligand from the protein‐bound fraction as previously described by us [118]. The radioligands (4–8 MBq) in a volume of 4 μL EtOH were added to 996 µL of plasma or PBS and mixed by repeated pipetting. Aliquots (250 μL) were loaded into the centrifugal filters and centrifuged at 14,000 *g* at 20°C for 30 min. After centrifugation, the filter and the bottom cup with the collected filtrate was each measured in a well‐type counter (ISOMED 2100, NUVIA Instruments GmbH, Dresden, Germany).

### 3.9 Stability Studies In Vitro

In vitro stability of **[** ^**123**^ **I]3** and **[** ^**123**^ **I]4** was analyzed after incubation of the radiotracer with IS, PBS, DMEM, and plasma. 15–25 MBq of the radiotracer in 20 µL EtOH were added to 380 µL of respective medium and incubated at 37°C (plasma, DMEM) and room temperature (18–20°C) (IS, PBS), respectively. Samples of 40 µL were withdrawn after distinct timepoints and either analyzed directly or after protein precipitation (DMEM and plasma). To this end, the aliquot of 40 µL was added to 160 µL of ice cold CH_3_CN. The mixture was vortexed for 30 s, stored on ice for 4 min, and centrifuged (5 min at 14,000 *g*, 4°C). Aliquots for analytical radio‐HPLC (100 µL, system 5, gradients 1 and 3) and radio‐TLC (2 µL, RP 60 % v/v CH_3_CN/H_2_O) were withdrawn from the supernatant.

Liver microsome experiments with **[** ^**123**^ **I]3** in the presence of NADPH (oxidizing conditions) were performed using Mouse (CD‐1) Microsomes (Gibco, Cat. No. MSMCPL, Lot MS053‐A) according to the procedure described by us with some modifications due to low stability of **[** ^**123**^ **I]3** in PBS [119]. Incubations had a final volume of 250 µL. The radiotracer prepared in the presence of Asc dissolved in ethanol (2 µL; 0.7–5.6 MBq/µL) was diluted with PBS (398 µL; 0.5 % v/v ethanol; 0.2 % v/v final) followed by the addition of DMSO (1 µL; no‐carrier–added mixture) or **3** (1 µL of 2.5 mM stock in DMSO, carrier–added mixture, 10 µM final). PBS (112.5 µL), mouse liver microsomes (12.5 µL of 20 mg/mL stock; 1 mg/mL final), and the radiotracer solution (100 µL) were mixed in a 1.5 mL Eppendorf tube and preincubated at 37°C for 5 min. Subsequently, NADPH (25 µL of a freshly prepared 20 mM solution in PBS, 2 mM final) was added and the mixture was further incubated at 37°C. After distinct time points (5, 10, 15, 30, and 60 min), an aliquot (40 µL) was withdrawn and subjected to protein precipitation as described before. Samples were analyzed using radio‐TLC (RP, 60 % v/v CH_3_CN/H_2_O). Testosterone (40 µM final concentration) in place of the radiotracer was used as positive control for oxidation. Complete conversion of testosterone was confirmed by HPLC‐DAD (system 3) after 60 min.

### 3.10 Cell Culture

The human glioblastoma cell line U87 MG (U87) (# HTB‐14) was purchased from ATCC (Manassas, VA, USA). The human colorectal adenocarcinoma cell line HT‐29 (ACC‐299) and the human acute monocytic leukemia cell line THP‐1 (ACC‐16) were purchased from DSMZ (Braunschweig, Germany). THP‐1 monocytes (MC) were differentiated into M0 macrophages (M*Φ*) using 64 nM PMA (Phorbol 12‐myristate 13‐acetate) for 3 days. Knockout of COX‐2 in U87 (U87^COX−2KO^) cells using CRISPR/Cas9 technology was performed as described previously [90]. All cells were cultured in Dulbecco´s modified Eagle´s medium (DMEM; U87, HT‐29) or Roswell Park Memorial Institute (RPMI; MC, M*Φ*) 1640 medium supplemented with 10 % v/v fetal calf serum (FCS) and 1 U/mL penicillin/streptomycin (all reagents from Biochrom, Berlin, Germany) under normoxic conditions (37°C, 5 % CO_2_).

### 3.11 Western Blot Analysis

SDS‐PAGE and Western blotting was performed as described previously [120]. PVDF membranes were incubated with anti‐COX‐2 (ab15919; 1:500, Abcam UK); anti‐5‐lipoxygenase (MA5−38 050; 1:2000, Thermo Scientific, Germany) (66326‐1‐Ig; 1:2000, Proteintech, Germany); anti‐β‐actin (A5060; 1:1000, Sigma–Aldrich, Germany) antibodies, followed by incubation with appropriate peroxidase‐coupled secondary antibody (anti‐rabbit IgG, A0545, Sigma–Aldrich, 1:5000; anti‐mouse IgG, A9044, Sigma–Aldrich, 1:10.000). Bands were visualized using SuperSignal West Pico and Femto chemiluminescent Substrate (Thermo Fisher Scientific, Germany) and imaged with an MF‐ChemiBIS Bio‐Imaging System (Biostep GmbH, Burkhardtsdorf, Germany). Western blot densitometry and relative intensity of protein bands were calculated using Aida 5.10 software.

### 3.12 Cell Uptake Studies

Radiotracer uptake studies were performed in confluent monolayer (U87, U87^COX−2KO^, HT‐29, M*Φ*) or in suspension (MC) as described elsewhere with some modifications [60, 61]. In brief, for monolayer experiments, cells were seeded in 24‐well plates at a density of 5 × 10^4^ cells/mL and grown to ≈ 80 % confluence. MC were centrifuged and resuspended in fresh RPMI medium containing 10 % FCS in 24‐well plates at a density of 5 × 10^5^ cells/mL. A solution of **[** ^**123**^ **I]3** and **[** ^**123**^ **I]4**, respectively, was added to the cells (200 µL per well, 0.1–0.9 MBq/mL, <0.1 % v/v EtOH content), and cellular binding and uptake was investigated after indicated timepoints at 37°C. For blocking experiments, cells were preincubated for 30 min with the respective blocking agent at 100 µM final concentration before addition of the radiotracer. The use of DMSO stock solutions resulted in a residual concentration of 1 % v/v DMSO content. Tracer uptake was stopped with 1 mL ice‐cold PBS, the cells were washed three times with PBS and dissolved in 0.5 mL NaOH (0.1 M containing 1 % w/v sodium dodecylsulfate). The radioactivity in the cell extracts was measured with a Wizard^2^ 2480 automatic gamma counter (PerkinElmer, Waltham, MA, USA). Total protein concentration in the samples was determined by the bicinchoninic acid method as described before [120]. Uptake data are expressed as percent initial dose per mg protein (%ID/mg protein).

### 3.13 Tumor Xenograft Models in Mice

All animal experiments were carried out according to the guidelines of the German Regulations for Animal Welfare and have been approved by the local Ethical Committee for Animal Experiments (reference number: DD24.1‐5131/499/49). General anesthesia was induced and maintained by inhalation of 10 % (v/v) desflurane in 30/70 % (v/v) oxygen/air. Animals were warmed during anesthesia at 37°C. Rj:NMRI‐*Foxn1* ^*nu/nu*^ mice (Janvier Labs, Le Genest‐Saint‐Isle, France) were xenotransplanted via subcutaneous injection of U87 glioblastoma cells (5 × 10^6^), HT‐29 colorectal carcinoma cells (3 × 10^6^), or THP‐1 monocytes MC (1 × 10^6^), each suspended in 100 µL Dulbecco´s PBS. The animal's general condition was monitored daily; body weight and tumor growth were monitored three times a week. Tumor size was measured by caliper, and tumor volume was calculated using the formula *V* = *π* / 6 × *abc*, assuming a triaxial ellipsoid with the axes *a*, *b*, and *c*. For urine collection, animals were allowed to roam separately in an empty, clean, conventional cage. After spontaneous miction, the urine was aspirated with a pipet tip and immediately used for further analysis. After the final experiment, anesthetized animals were sacrificed via cervical dislocation. Tissue explants were prepared and used for further analysis.

### 3.14 Quantitative SPECT Imaging

Animals were included in imaging studies when tumors reached a diameter > 7 mm. Tumor‐bearing mice (body weight 29.7 ± 3.7 g) received an intravenous injection of **[** ^**123**^ **I**]**3** (22.6 ± 3.8 MBq) or **[** ^**123**^ **I]4** (18.5 ± 6.7 MBq) into a lateral tail vein, each delivered in 200 µL PBS. SPECT imaging of anesthetized animals (desflurane) was performed using the nanoScan SPECT/CT (Mediso Medical Imaging Systems, Budapest, Hungary), equipped with the APT56 aperture consisting of four multipinhole ultrahigh energy collimators. Photon emission was recorded starting 1 h and 4 h after radiotracer injection, each with a scanning time of 30 min. With each scan, a corresponding CT image was captured and used for anatomical referencing and attenuation correction. Emission data were binned within the 20% energy window of the 159 keV photopeak and reconstructed using the Tera‐Tomo 3D algorithm with a voxel size of 0.23 mm applying corrections for attenuation, scattering, collimator plate scattering, and decay. Images were analyzed using Rover version 3.0.80h (ABX GmbH). Three‐dimensional regions of interest were generated within spherical preselection masks including voxels with intensities above tissue‐specific thresholds (% of maximum voxel intensity) as follows: gall bladder and intestine (>10%), stomach (> 10%), thyroid and salivary glands (> 2%), urinary bladder (> 10%). ROI‐specific standardized uptake values (SUVtotal) were extracted and converted into percent of the initially administered radiotracer dose (% ID). Maximum‐intensity projections of SPECT/CT overlays were generated using InterView Fusion version 3.09 (Mediso) and presented with common scale.

### 3.15 Metabolite Analysis in Urine

To investigate the stability of **[** ^**123**^ **I]3** and **[** ^**123**^ **I]4** in mice, urine samples were collected 1.5 h, 4.5 h, and 24 h after injection (after SPECT scanning, *n* = 1 for each radiotracer). 10 µL of the urine sample were mixed with 20 µL of 15% v/v trichloroacetic acid (TCA) in water, vortexed for 10 s and centrifuged (5 min, 14,000 *g*, 4°C). A sample for radio‐TLC was withdrawn from the clear supernatant (1 µL) and analyzed on RP‐TLC using 60% v/v CH_3_CN/H_2_O.

## 4 Conclusion

COX‐2 and 5‐LO have been identified as promising biological targets for the development of radiotracers for noninvasive characterization of cancer and inflammatory diseases. The implementation of carborane moieties into organic dual inhibitor molecules of these inflammation markers may lead to the generation of a new class of pharmaceuticals with minimized side effects. In the context of radiotracer synthesis, the carborane cluster conveys metabolic stability to the tracer molecules, which accommodates for long biological half‐lives, with the half‐life of radioisotope iodine‐123 being ideal for this application. We found that iodination of the RWJ‐63556‐based carborane analogs **1** and **2** led to a decreased inhibition potency both toward COXs and 5‐LO. While selectivity of **3** and **4** for COX‐2 over COX‐1 is retained, the iodinated compounds were only moderately potent COX‐2 inhibitors. However, inhibitory potency toward 5‐LO remained in the sub‐micromolar range. Radioiodination and formulation with antioxidants provided solutions of the radiotracers suitable for in vitro and in vivo radiopharmacological studies, although precautions in aqueous media had to be considered due to tendency of the tracers for radiodeiodination. Cellular uptake of **[** ^**123**^ **I]3** and **[** ^**123**^ **I]4** proved to be independent of COX‐2 while for 5‐LO, the uptake studies indicated for selected tumor cell lines partly 5‐LO mediated uptake, but not for inflammatory cells. However, xenograft mouse models finally revealed that no tumor accumulation could be observed in different tumor models characterized for their 5‐LO and COX‐2 expression. Further, the radiotracer showed fast excretion of activity via the urine likely caused by hepatic radiodeiodination as main pathway in phase‐1 metabolism. These results render **[** ^**123**^ **I]3** and **[** ^**123**^ **I]4** not suitable as radiotracers for imaging of COX‐2 or 5‐LO. Nevertheless, importance of both targets in cancer—also reflected by the expression patterns in the different tumor models investigated in this study—warrants future efforts in tracer development.

## Supporting Information

The authors have cited additional references within the Supporting Information [58, 59, 111]. Additional supporting information can be found online in the Supporting Information section. **Supporting Fig. S1:** Reaction control of iodination of compound **1**. Sample withdrawn at 60 min. Peaks in order from left to right: **1**, Cl‐**1**, **3.** **Supporting Fig. S2:** m/z of Cl‐**1** extracted from reaction control depicted in Figure S 18. **Supporting Fig. S3:** Reaction control after 30 min **4**; Peaks in order from left to right: **2**, Cl‐**2**; **4.** **Supporting Fig. S4:** ^1^H NMR of **3** in CDCl_3_. **Supporting Fig. S5:** ^11^B NMR of **3** in CDCl_3_. **Supporting Fig. S6:** ^11^B{^1^H} NMR of **3** in CDCl_3_. **Supporting Fig. S7:** 13C NMR of **3** in CDCl_3_ . **Supporting Fig. S8:** H,H‐COSY of **3** in CDCl_3_. **Supporting Fig. S9:** NOESY of **3** in CDCl_3_. **Supporting Fig. S10:** HSQC of **3** in CDCl_3_. **Supporting Fig. S11:** HMBC of **3** in CDCl_3_. **Supporting Fig. 12:** ^1^H NMR **4** in (CD3)2SO. **Supporting Fig. S13:** ^11^B NMR **4** in CDCl_3_. **Supporting Fig. S14:** ^11^B{^1^H} **4** CDCl_3_. **Supporting Fig. S15:** 13C NMR of **4** in (CD3)2SO. **Supporting Fig. S16:** H,H‐COSY of **4** in (CD3)2SO. **Supporting Fig. S17:** NOESY of **4** in (CD3)2SO. **Supporting Fig. S18:** HSQC of **4** in (CD3)2SO. **Supporting Fig. S19:** HMBC of **4** in (CD3)2SO. **Supporting Fig. S20:** HPLC purity analysis of **3** (system 5, gradient 2). **Supporting Fig. S21:** HR‐MS of **3** (HR‐MS1, ESI‐). **Supporting Fig. S22:** HPLC purity analysis **4** (system 5, gradient 3). **Supporting Fig. S23:** HR‐MS of **4** (HR‐MS2, ESI‐). **Supporting Fig. S24:** COX inhibition as determined using COX Fluorescent Inhibitor Screening Assay Kit. Left column COX‐1, right column COX‐2. Top row **3**, bottom row **4**. **Supporting Fig. S25:** 5‐LO inhibition in whole cell PMNL assay by **3** (JS 61/23) and **4** (JS 01/24). **Supporting Fig. S26:** Optimization of radioiodination conditions of **1** in the presence of chloramine‐T (CAT). Left: successive dilution of **1** and CAT. Below 100 μM, RCC harshly decreases. Right: Increasing concentrations of CAT reestablish RCC up to 60 %, but initial values of > 80 % are not achieved. **Supporting Fig. S27:** **[** ^**123**^ **I]3** coinjected with **3** (system 5, gradient 1). **Supporting Fig. S28:** **[** ^**123**^ **I]4** coinjected with **4** (system 5, gradient 1). **Supporting Fig. S29:** Incubation of **[** ^**123**^ **I]3** in the presence of different antioxidants. **Supporting Fig. S30:** Stability of **[** ^**123**^ **I]4** in the presence of antioxidants Asc and GA. **Supporting Fig. S31:** Stability of **[** ^**123**^ **I]3** and **[** ^**123**^ **I]4** in DMEM and human plasma filtrate in the presence of Asc. **Supporting Fig. S32:** Radio‐TLC following murine liver microsome assay of **[** ^**123**^ **I]3**. Samples withdrawn after 5 min (a), 10 min (b), 15 min (c), 30 min (d), and 60 min (e). Sample of tracer incubation with added non‐radioactive reference substance **3** (final concentration 10 μM; f). Sample of tracer incubation under control conditions (without added NADPH; g). **Supporting Fig. S33**: Western blot analysis with 5‐LO antibody (A, B, C) and COX‐2 antibody (D). Bottom row: loading control with β‐actin. (A) Whole cell lysates, NMRI*nu*/*nu* lung tissue served as positive control for 5‐LO. (B) Fractionated cell lysates from cytoplasm and nucleus. (C) Tumor lysates after explantation from xenografted NMRI*nu*/*nu* mice with NMRI*nu*/*nu* lung tissue as positive control for 5‐LO. (D) Tumor lysates after explantation from xenografted NMRInu/nu mice with mouse pheochromocytoma cells as positive control for COX‐2. Samples are labelled as follows: (1) NMRI*nu*/*nu* lung tissue, (2) U87 cells or tumor, (3) U87COX‐2KO cells, (4) HT‐29 cells or tumor, (5) MC cells or tumor, (6) MΦ cells, (7) mouse pheochromocytoma cells. **Supporting Fig. S34**: Cell uptake studies of **[** ^**123**^ **I]3**. **Supporting Fig. S35**: Cell uptake studies of **[** ^**123**^ **I]4**. **Supporting Fig. S36:** Distribution of **[** ^**123**^ **I]3** and **[** ^**123**^ **I]4** in mice visualized using quantitative SPECT imaging. Maximum‐intensity projections U87, HT‐29, or THP‐1 tumor‐bearing mice at indicated time points after intravenous injection of the radiolabeled compounds (n.c.a), each administered at an initial dose of 20 MBq in presence of ascorbic (Asc) acid or gentisic acid (GA) as antioxidant. (SUV) standardized uptake value. **Supporting Fig. S37**: Distribution of **[** ^**123**^ **I]4** in a U87 xenografted mouse visualized using quantitative SPECT imaging as reported earlier.116 Maximum‐intensity projections at indicated time points after intravenous injection of the radiolabeled compound (n.c.a), administered at an initial dose of 20 MBq in presence of Asc as antioxidant. An accumulation of activity into the marginal region of the tumor was observed over the course of 4 h, which was retained over 24 h. This effect only occurred in this individual case and could not be replicated in subsequent experiments. The specific tumor was not explanted and therefore could not be further characterized regarding enzyme expression and cellular composition. The accumulation in the highly COX‐2 expressing periphery of HCA‐7 xenografts was reported for other COX‐2 radiotracers.64,125 However, because this pattern did not reappear, it is treated as an isolated, possibly non‐specific event with uncertain biological relevance. **Supporting Fig. S38:** Radio‐TLC of urine samples collected 1.5 h, 4.5 h, and 24 h after injection of **[** ^**123**^ **I]3** and **[** ^**123**^ **I]4**, respectively. (a) reference compound, (b) sample after protein precipitation using 15 % v/v TCA, (c) sample without protein precipitation. **Supporting Table S1:** RCY, RCP and *A*_M_ of **[** ^**123**^ **I]3** in the presence of antioxidants (none, Asc, GA).

## Funding

This study was supported by Deutsche Forschungsgemeinschaft (Grant HE 1376/54‐1 and PI 304/7‐1).

## Conflicts of Interest

The authors declare no conflicts of interest.

## Supporting information

Supplementary Material

## Data Availability

The data that support the findings of this study are available from the corresponding author upon reasonable request.
