# Supplementary material for: Radioiodination of Two Carborane‐Based Dual Cyclooxygenase‐2/5‐Lipoxygenase Inhibitors and Their In Vitro and In Vivo Evaluation
Source: Chembiochem. 2026 Mar 31;27(7):e202600004. doi: 10.1002/cbic.202600004 (PMC13039775; doi:10.1002/cbic.202600004)
Supplement: Supplementary file 1 — Supplementary Material [file CBIC-27-e202600004-s001.pdf]

# Supporting information

## Table of Contents

Synthesis of **3** and **4**.......... 3

Spectra of **3** and **4**.......... 5

Assays .......... 15

COX Assay .......... 15

5-LO Assay .......... 15

Radiolabeling .......... 16

Stability of Radiotracers **[$^{123}$I]3** and **[$^{123}$I]4** .......... 18

Western blot images .......... 19

Cell uptake studies .......... 20

SPECT images.......... 22

Metabolite analysis in urine .......... 24

Figure S 1 Reaction control of iodination of compound **1**. Sample withdrawn at 60 min. Peaks in order from left to right: **1**, Cl-**1**, **3**.......... 3
Figure S 2 m/z of Cl-**1** extracted from reaction control depicted in Figure S 18. .......... 3
Figure S 3 Reaction control after 30 min **4**; Peaks in order from left to right: **2**, Cl-**2**; **4**.......... 4
Figure S 4 $^{1}$H NMR of **3** in $CDCl_3$.......... 5
Figure S 5 $^{11}$B NMR of **3** in $CDCl_3$.......... 5
Figure S 6 $^{11}$B{$^{1}$H} NMR of **3** in $CDCl_3$ .......... 6
Figure S 7 $^{13}$C NMR of **3** in $CDCl_3$.......... 6
Figure S 8 H,H-COSY of **3** in $CDCl_3$.......... 7
Figure S 9 NOESY of **3** in $CDCl_3$ .......... 7
Figure S 10 HSQC of **3** in $CDCl_3$ .......... 8
Figure S 11 HMBC of **3** in $CDCl_3$ .......... 8
Figure S 12 $^{1}$H NMR **4** in $(CD_3)_2SO$.......... 9
Figure S 13 $^{11}$B NMR **4** in $CDCl_3$.......... 9
Figure S 14 $^{11}$B{$^{1}$H} **4** $CDCl_3$ .......... 10
Figure S 15 $^{13}$C NMR of **4** in $(CD_3)_2SO$ .......... 10
Figure S 16 H,H-COSY of **4** in $(CD_3)_2SO$ .......... 11
Figure S 17 NOESY of **4** in $(CD_3)_2SO$ .......... 11
Figure S 18 HSQC of **4** in $(CD_3)_2SO$ .......... 12
Figure S 19 HMBC of **4** in $(CD_3)_2SO$ .......... 12
Figure S 20 HPLC purity analysis of **3** (system 5, gradient 2).......... 13
Figure S 21 HR-MS of **3** (HR-MS1, ESI$^-$) .......... 13
Figure S 22 HPLC purity analysis **4** (system 5, gradient 3).......... 14
Figure S 23 HR-MS of **4** (HR-MS2, ESI$^-$) .......... 14
Figure S 24 COX inhibition as determined using COX Fluorescent Inhibitor Screening Assay Kit. Left column COX-1, right column COX-2. Top row **3**, bottom row **4**. .......... 15
Figure S 25 5-LO inhibition in whole cell PMNL assay by **3** (JS 61/23) and **4** (JS 01/24). .......... 15

Figure S 26 Optimization of radioiodination conditions of **1** in the presence of chloramine-T (CAT). Left: successive dilution of **1** and CAT. Below 100 µM, RCC harshly decreases. Right: Increasing concentrations of CAT reestablish RCC up to 60 %, but initial values of > 80 % are not achieved...... 16
Figure S 27 **[$^{123}$I]3** coinjected with **3** (system 5, gradient 1)............................................................ 16
Figure S 28 **[$^{123}$I]4** coinjected with **4** (system 5, gradient 1)............................................................ 16
Figure S 29 Incubation of **[$^{123}$I]3** in the presence of different antioxidants. ...................................... 17
Figure S 30 Stability of **[$^{123}$I]4** in the presence of antioxidants Asc and GA. ...................................... 18
Figure S 31 Stability of **[$^{123}$I]3** and **[$^{123}$I]4** in DMEM and human plasma filtrate in the presence of Asc. ........................................................................................................................................ 18
Figure S 32 Radio-TLC following murine liver microsome assay of **[$^{123}$I]3**. Samples withdrawn after 5 min (a), 10 min (b), 15 min (c), 30 min (d), and 60 min (e). Sample of tracer incubation with added non-radioactive reference substance **3** (final concentration 10 µM; f). Sample of tracer incubation under control conditions (without added NADPH; g). ...................................................................... 18
Figure S 33 Western blot analysis with 5-LO antibody (A, B, C) and COX-2 antibody (D). Bottom row: loading control with β-actin. (A) Whole cell lysates, NMRI$^{nu/nu}$ lung tissue served as positive control for 5-LO. (B) Fractionated cell lysates from cytoplasm and nucleus. (C) Tumor lysates after explantation from xenografted NMRI$^{nu/nu}$ mice with NMRI$^{nu/nu}$ lung tissue as positive control for 5-LO. (D) Tumor lysates after explantation from xenografted NMRInu/nu mice with mouse pheochromocytoma cells as positive control for COX-2. Samples are labelled as follows: (1) NMRI$^{nu/nu}$ lung tissue, (2) U87 cells or tumor, (3) U87$^{COX\text{-}2KO}$ cells, (4) HT-29 cells or tumor, (5) MC cells or tumor, (6) MΦ cells, (7) mouse pheochromocytoma cells. ............................................................................ 19
Figure S 34 Cell uptake studies of **[$^{123}$I]3**. ......................................................................................... 20
Figure S 35 Cell uptake studies of **[$^{123}$I]4**. ......................................................................................... 21
Figure S 36: Distribution of **[$^{123}$I]3** and **[$^{123}$I]4** in mice visualized using quantitative SPECT imaging. Maximum-intensity projections U87, HT-29, or THP-1 tumor-bearing mice at indicated time points after intravenous injection of the radiolabeled compounds (n.c.a), each administered at an initial dose of 20 MBq in presence of ascorbic (Asc) acid or gentisic acid (GA) as antioxidant. (SUV) standardized uptake value ........................................................................................... 22
Figure S 37 Distribution of **[$^{123}$I]4** in a U87 xenografted mouse visualized using quantitative SPECT imaging as reported earlier.[116] Maximum-intensity projections at indicated time points after intravenous injection of the radiolabeled compound (n.c.a), administered at an initial dose of 20 MBq in presence of Asc as antioxidant. An accumulation of activity into the marginal region of the tumor was observed over the course of 4 h, which was retained over 24 h. This effect only occurred in this individual case and could not be replicated in subsequent experiments. The specific tumor was not explanted and therefore could not be further characterized regarding enzyme expression and cellular composition. The accumulation in the highly COX-2 expressing periphery of HCA-7 xenografts was reported for other COX-2 radiotracers.[64,125] However, because this pattern did not reappear, it is treated as an isolated, possibly non-specific event with uncertain biological relevance. .................... 23
Figure S 38 Radio-TLC of urine samples collected 1.5 h, 4.5 h, and 24 h after injection of **[$^{123}$I]3** and **[$^{123}$I]4**, respectively. (a) reference compound, (b) sample after protein precipitation using 15 % v/v TCA, (c) sample without protein precipitation. ..................................................................... 24

Table S 1 RCY, RCP and $A_M$ of **[$^{123}$I]3** in the presence of antioxidants (none, Asc, GA). ....................... 17
Table S 2 RCY, RCP and $A_M$ of **[$^{123}$I]4** in the presence of antioxidants (Asc, GA). ................................ 17

# Synthesis of **3** and **4**

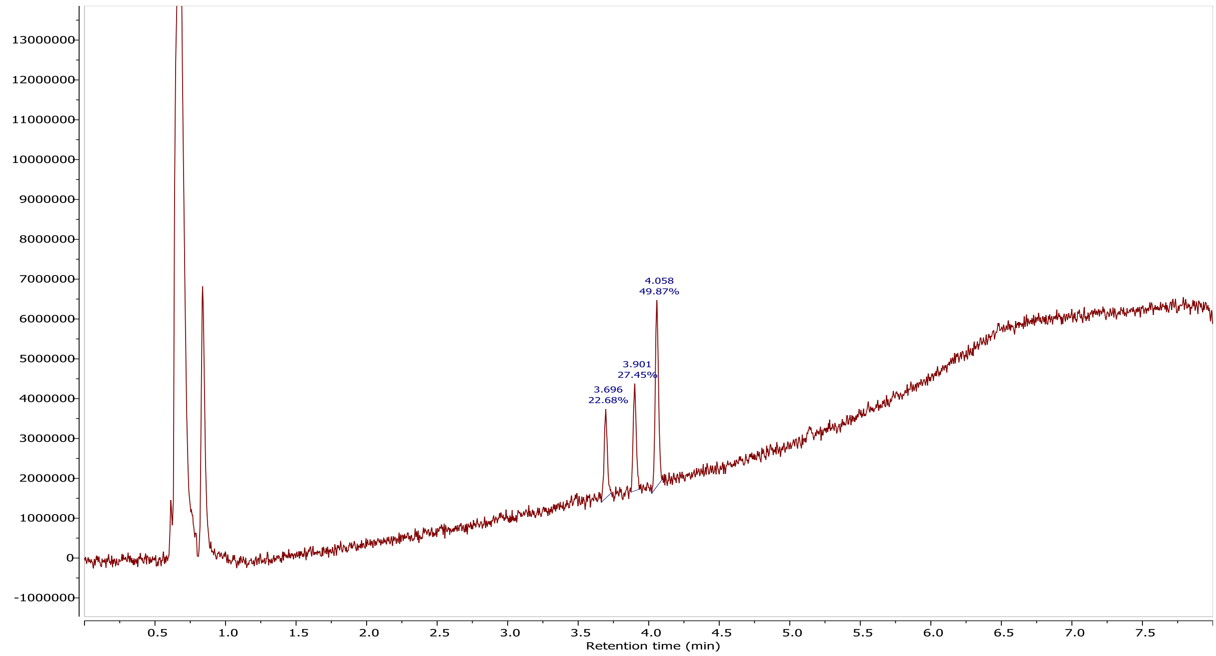


Figure S 1 Reaction control of iodination of compound **1**. Sample withdrawn at 60 min. Peaks in order from left to right: **1**, Cl-**1**, **3**

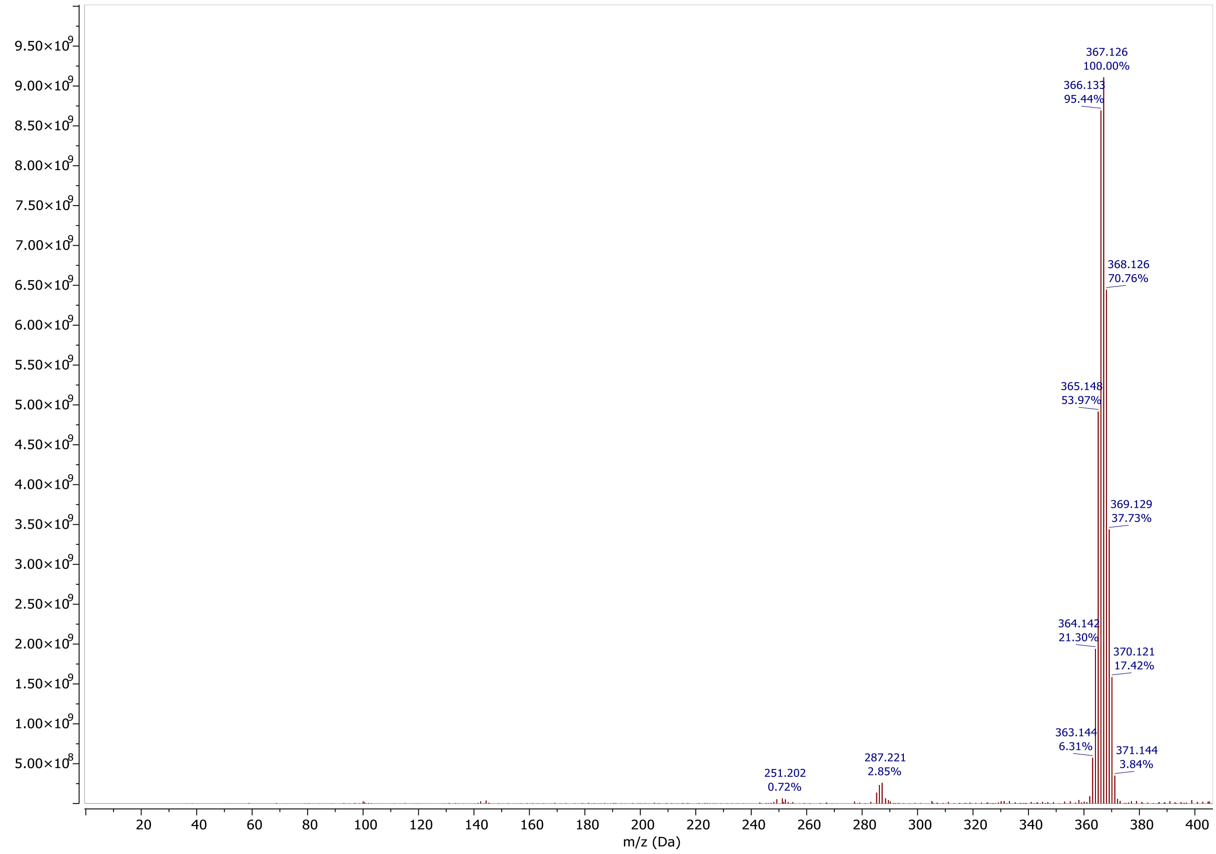


Figure S 2 m/z of Cl-**1** extracted from reaction control depicted in Figure S 18.

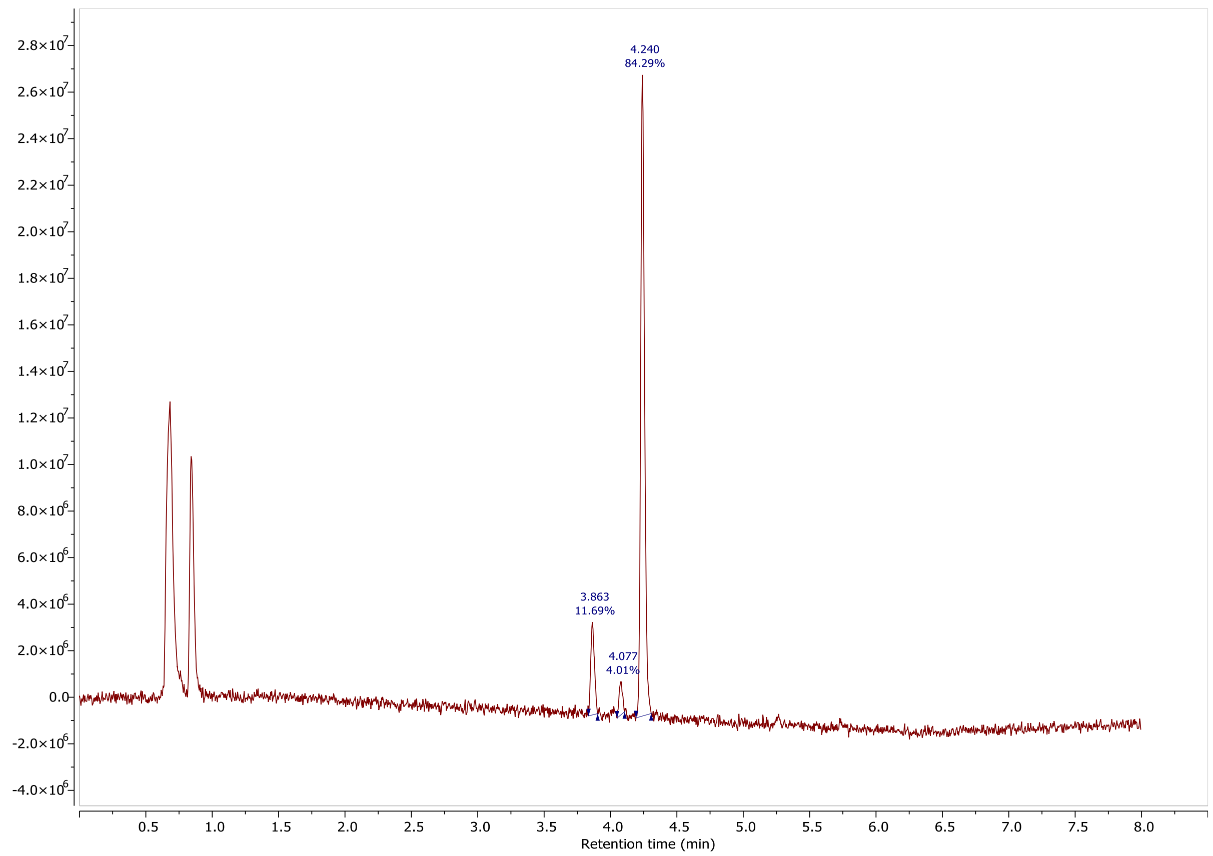


Figure S 3 Reaction control after 30 min **4**; Peaks in order from left to right: **2**, Cl-**2**; **4**

# Spectra of **3** and **4**

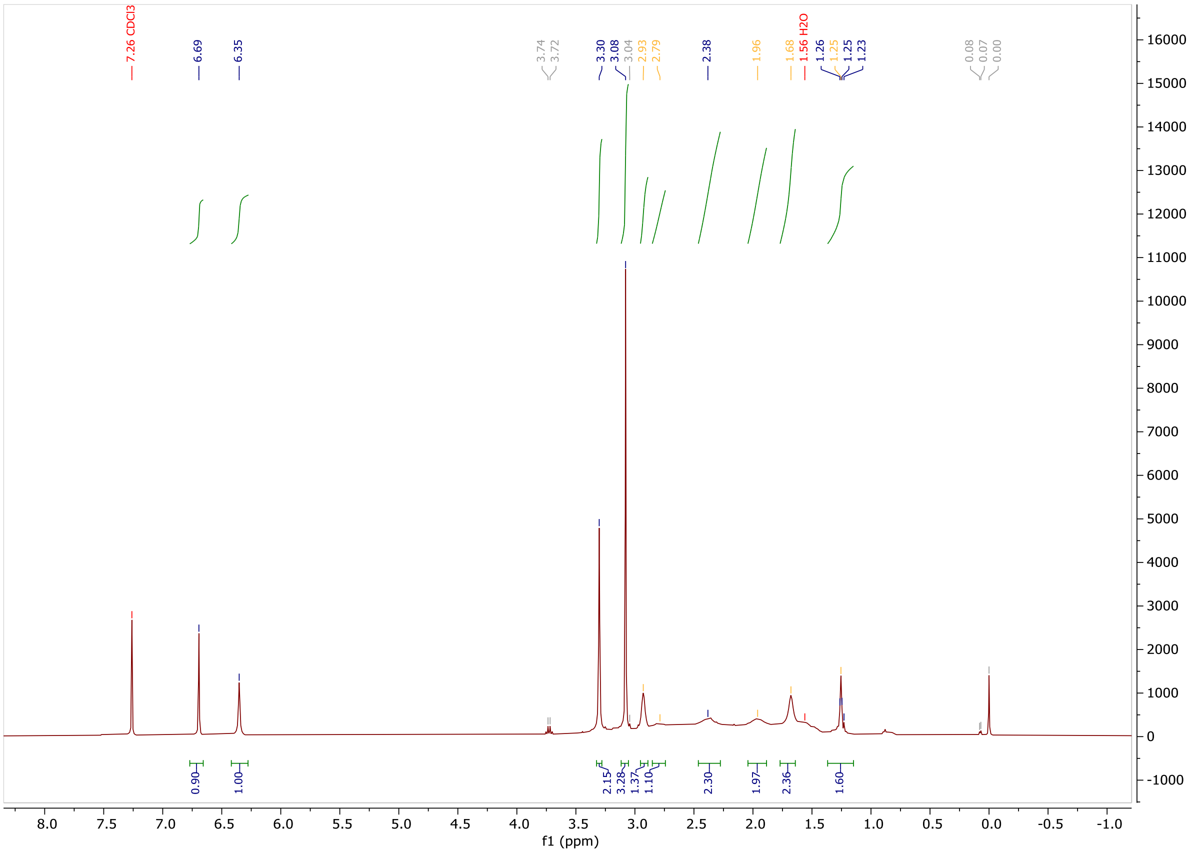


Figure S 4 $^{1}H$ NMR of **3** in $CDCl_3$

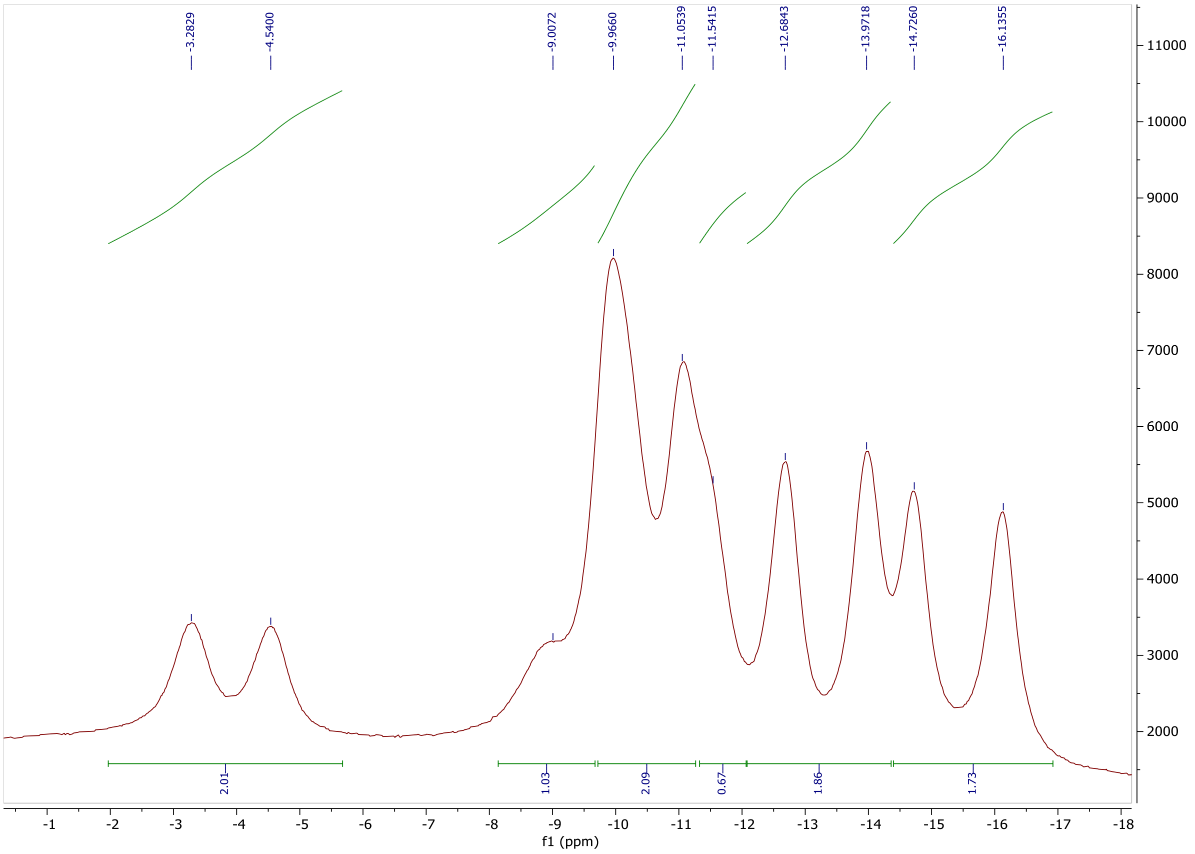


Figure S 5 $^{11}B$ NMR of **3** in $CDCl_3$

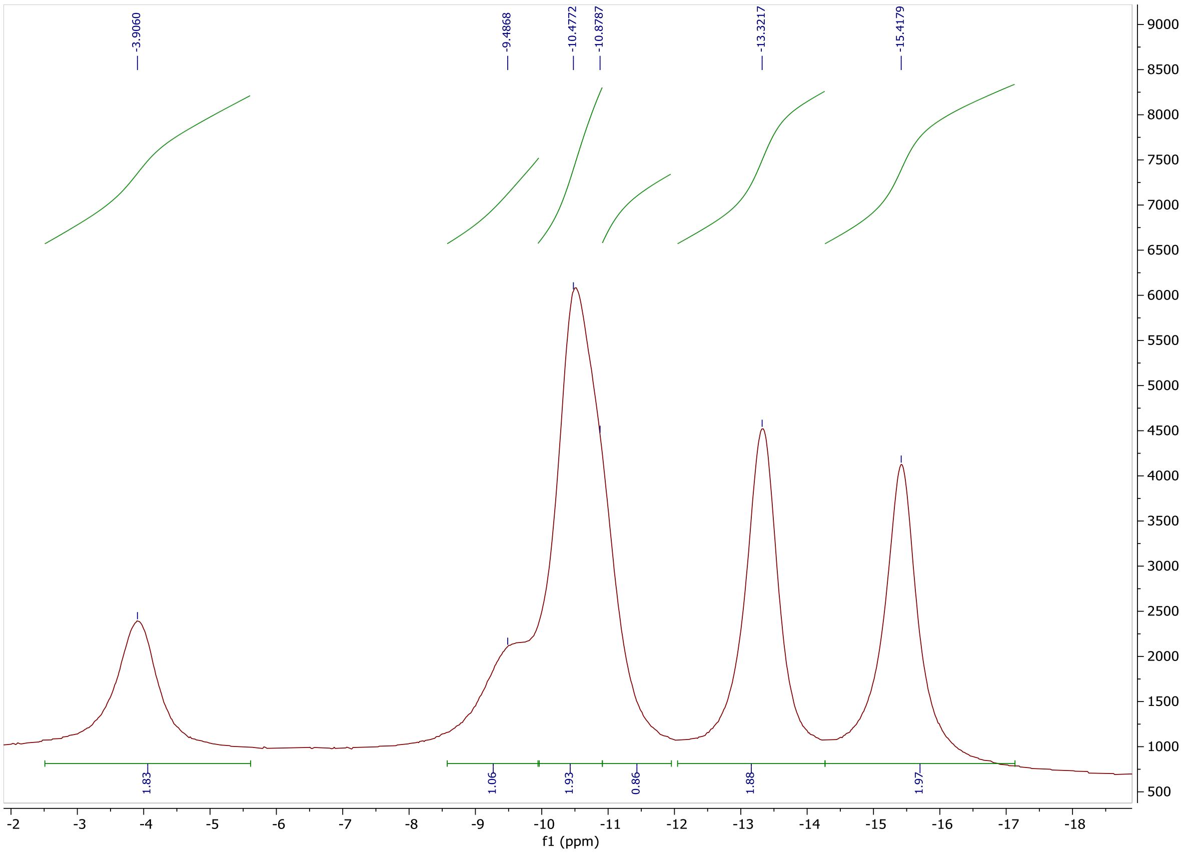


Figure S 6 $^{11}B\{^{1}H\}$ NMR of **3** in $CDCl_3$

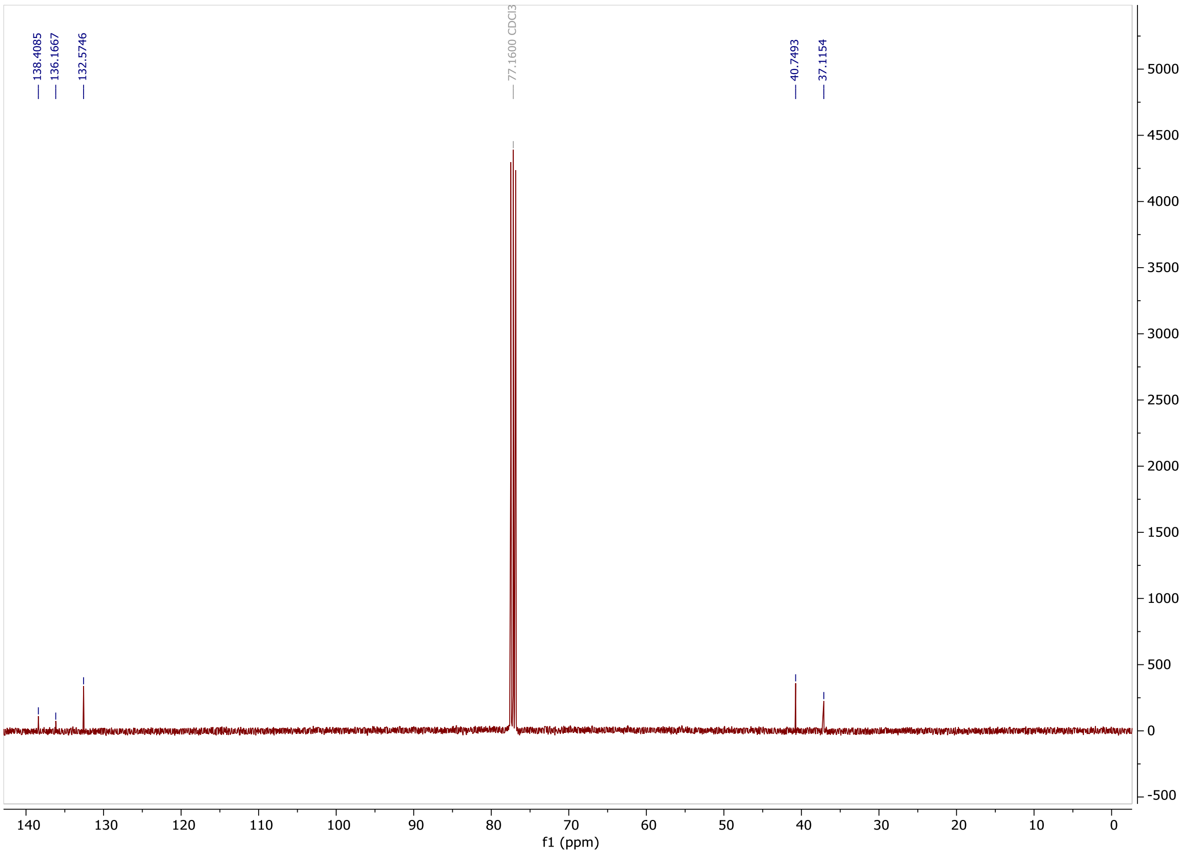


Figure S 7 $^{13}C$ NMR of **3** in $CDCl_3$

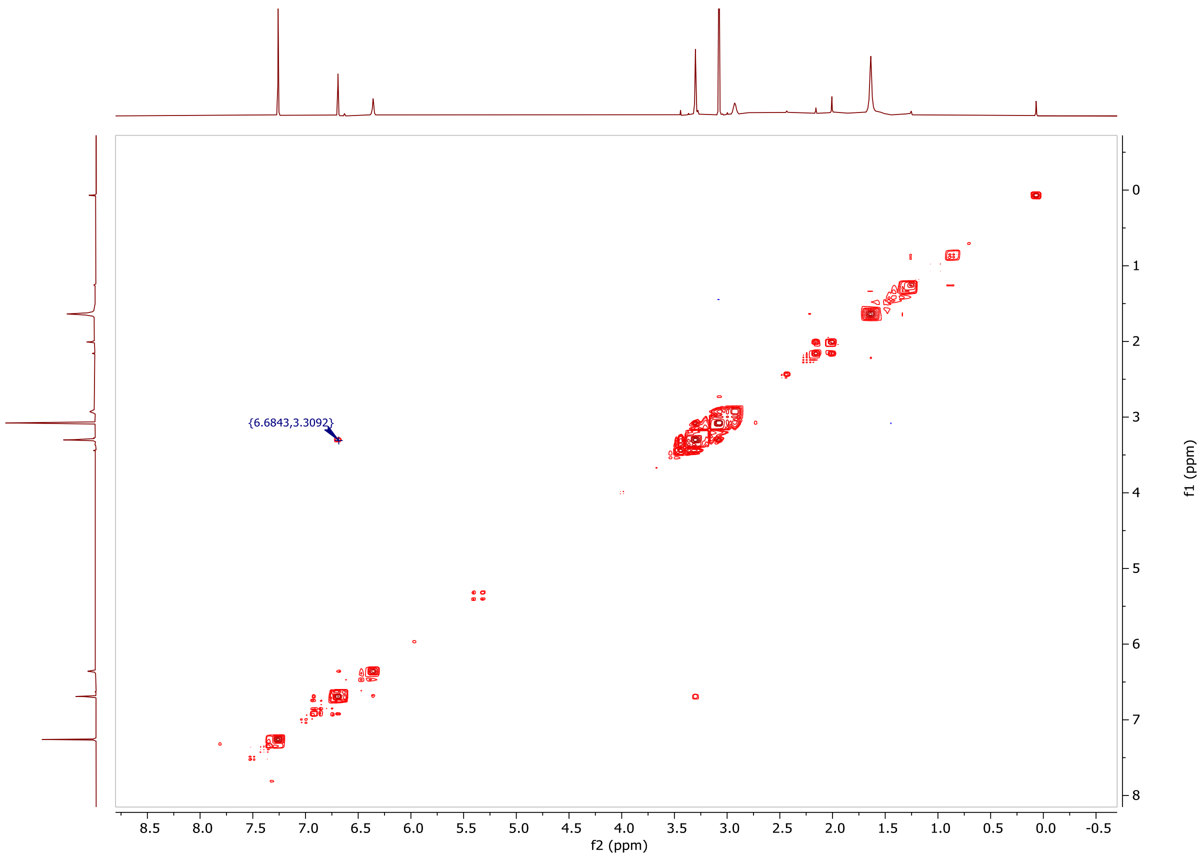


Figure S 8 H,H-COSY of **3** in $CDCl_3$

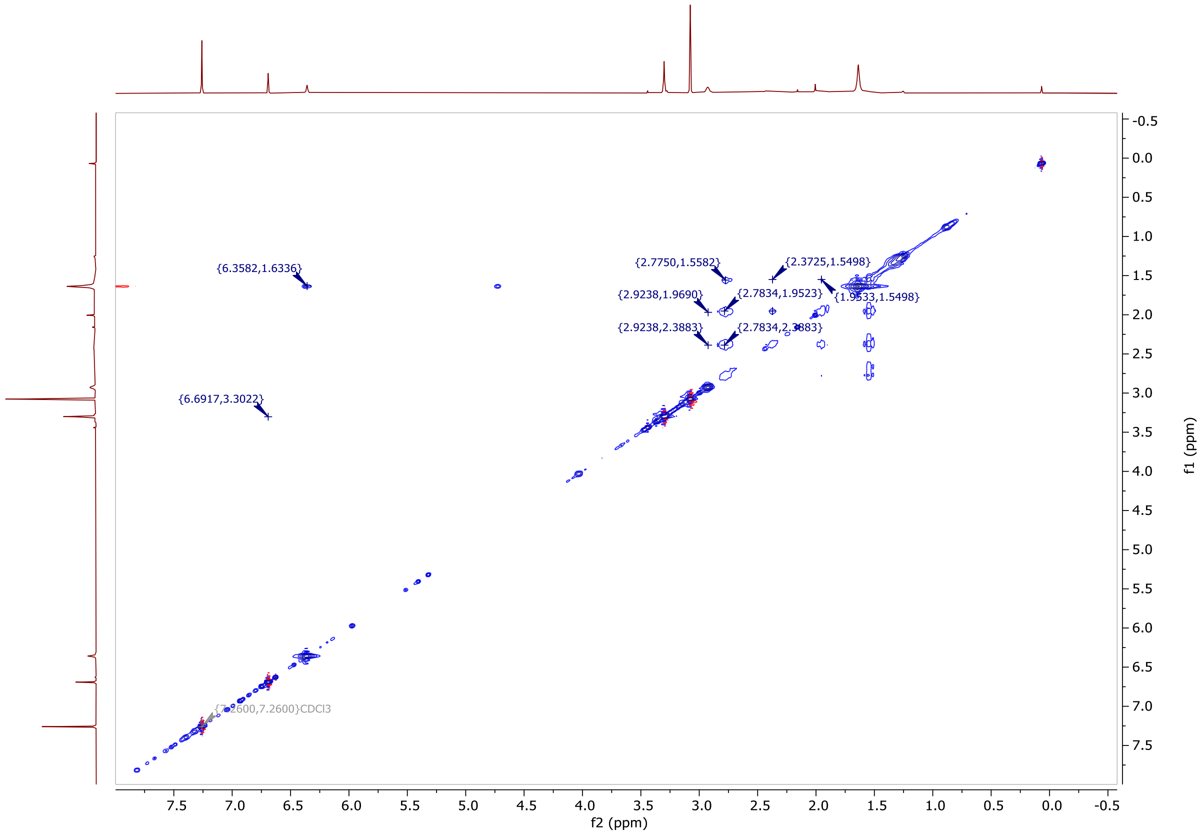


Figure S 9 NOESY of **3** in $CDCl_3$

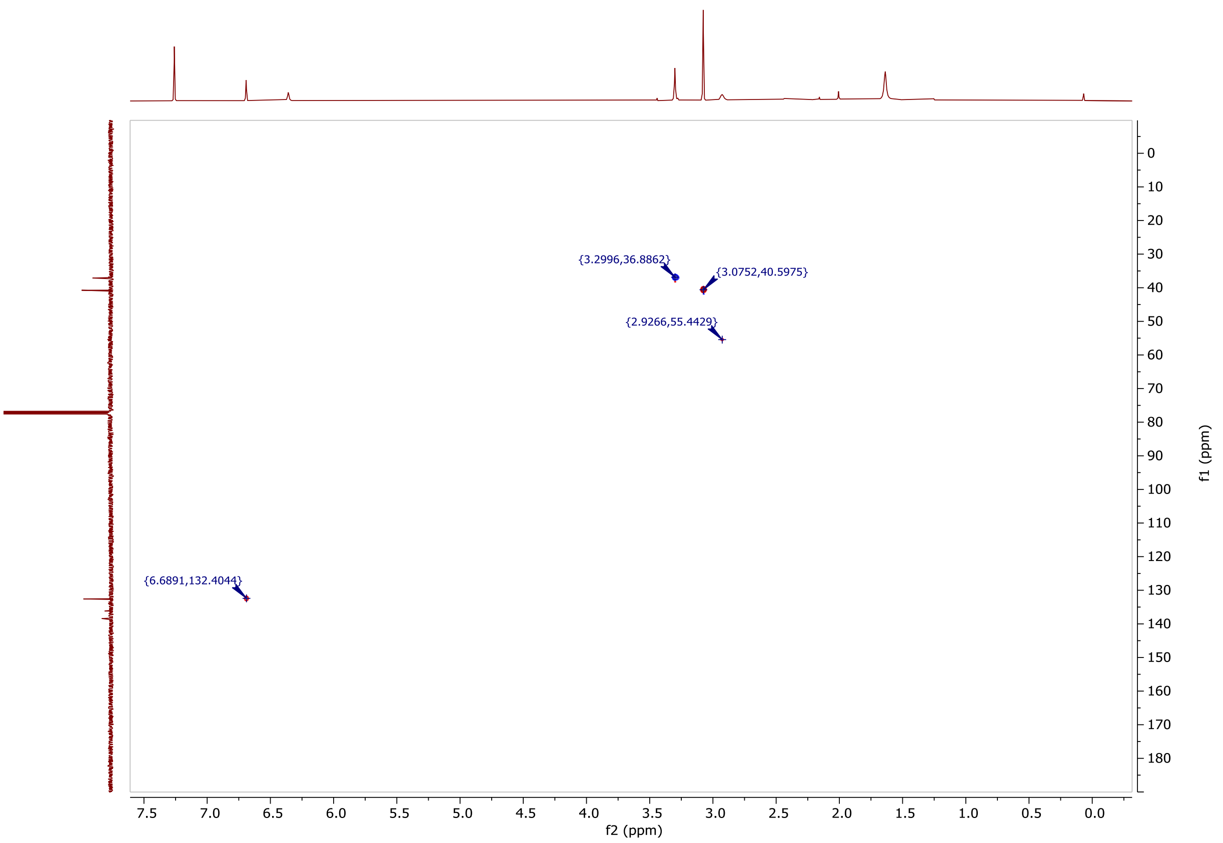


Figure S 10 HSQC of **3** in $CDCl_3$

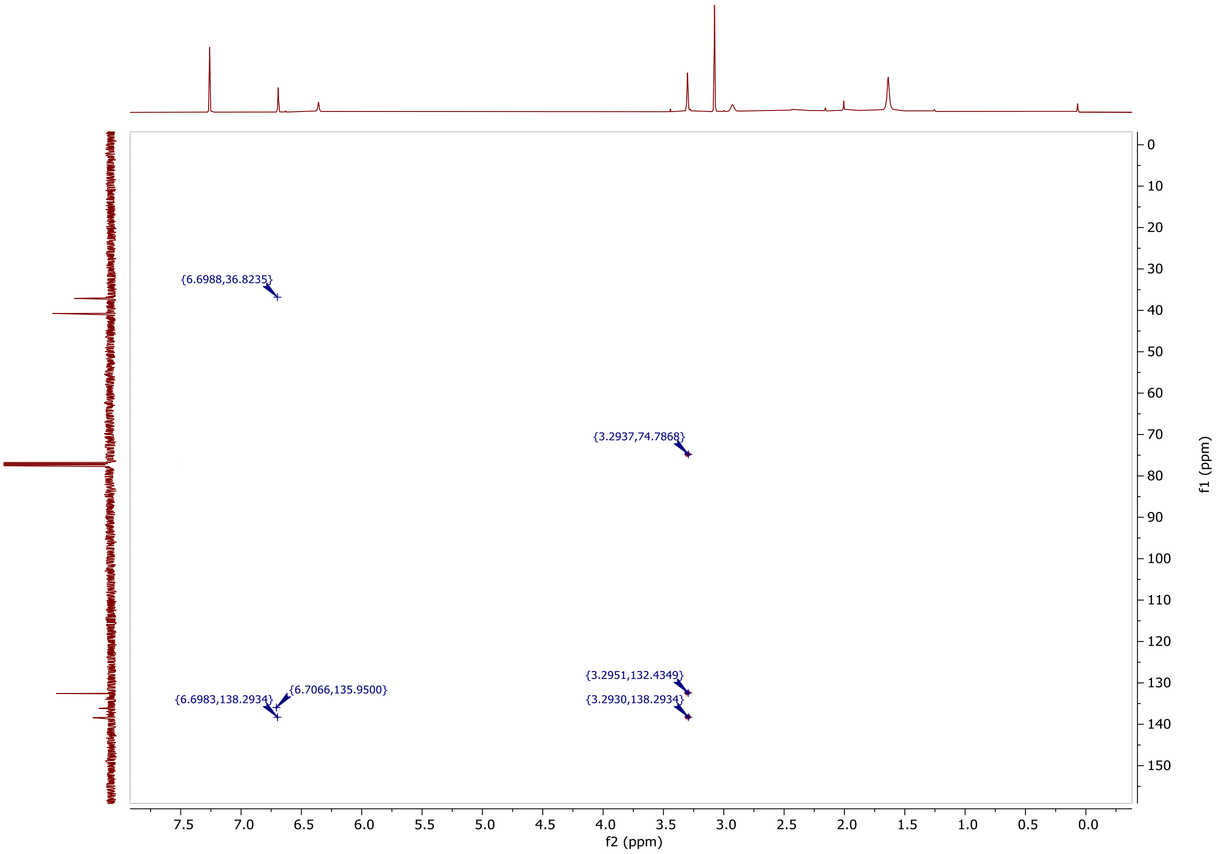


Figure S 11 HMBC of **3** in $CDCl_3$

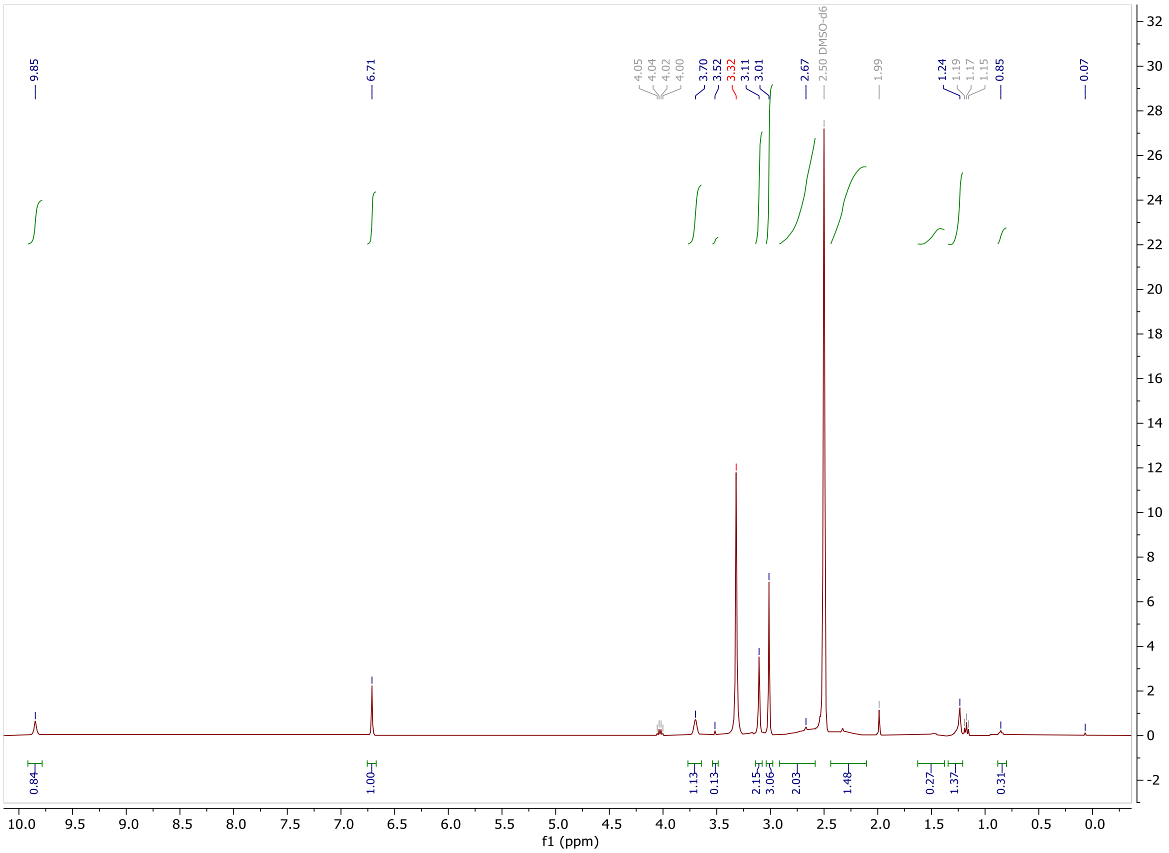


Figure S 12 $^{1}H$ NMR **4** in $(CD_3)_2SO$

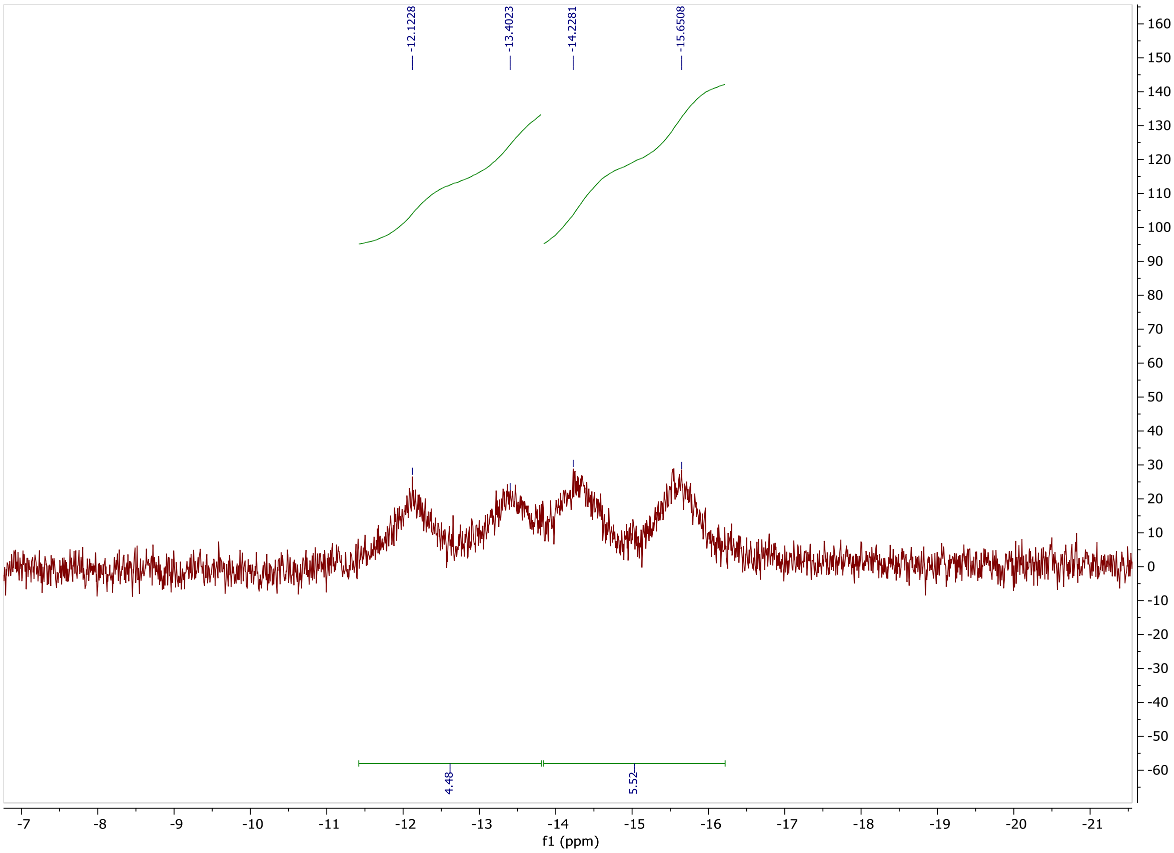


Figure S 13 $^{11}B$ NMR **4** in $CDCl_3$

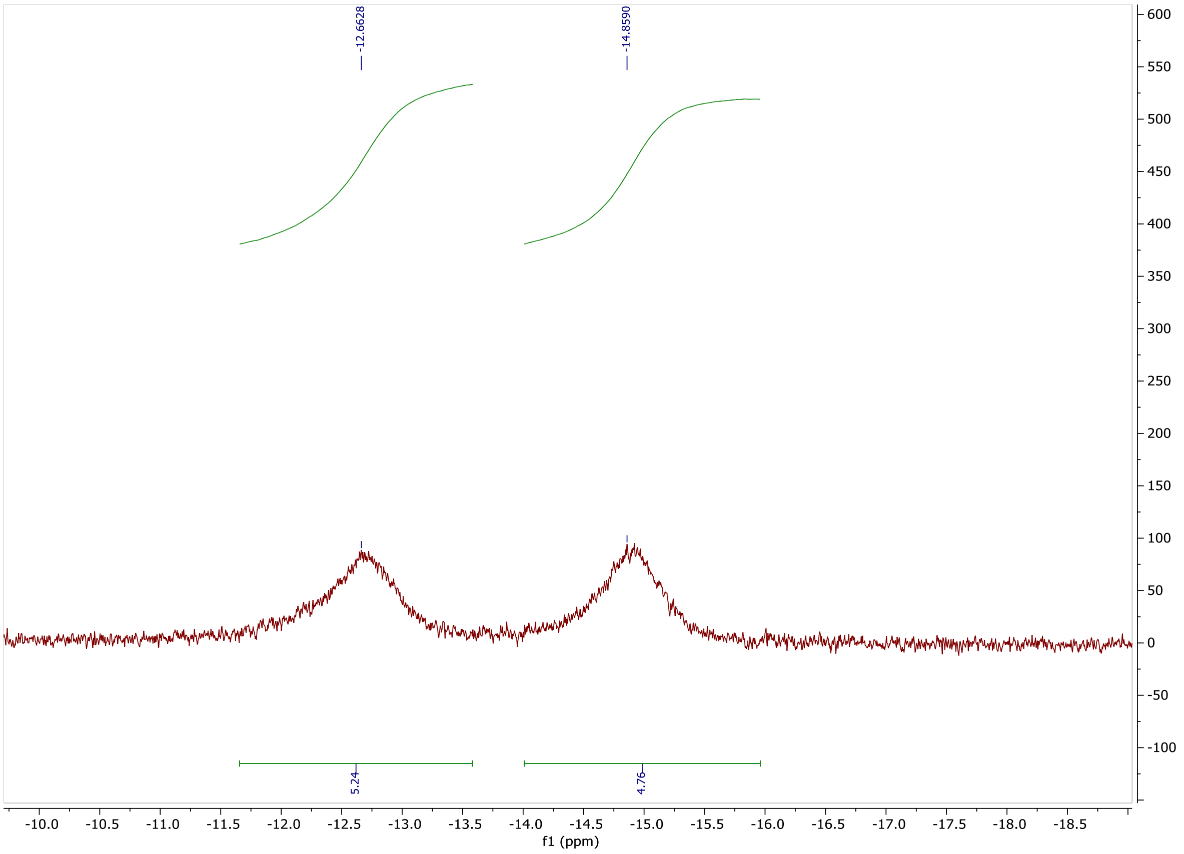


Figure S 14 $^{11}B\{^{1}H\}$ **4** $CDCl_3$

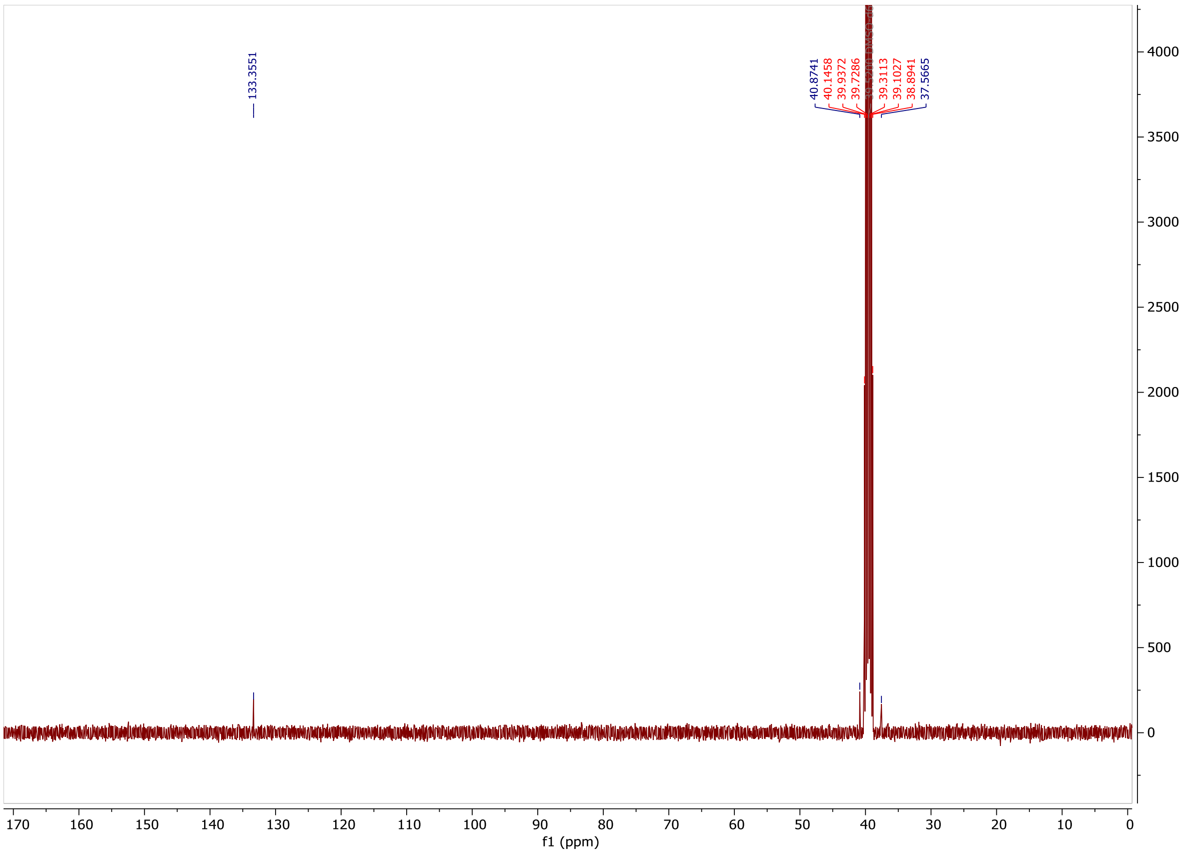


Figure S 15 $^{13}C$ NMR of **4** in $(CD_3)_2SO$

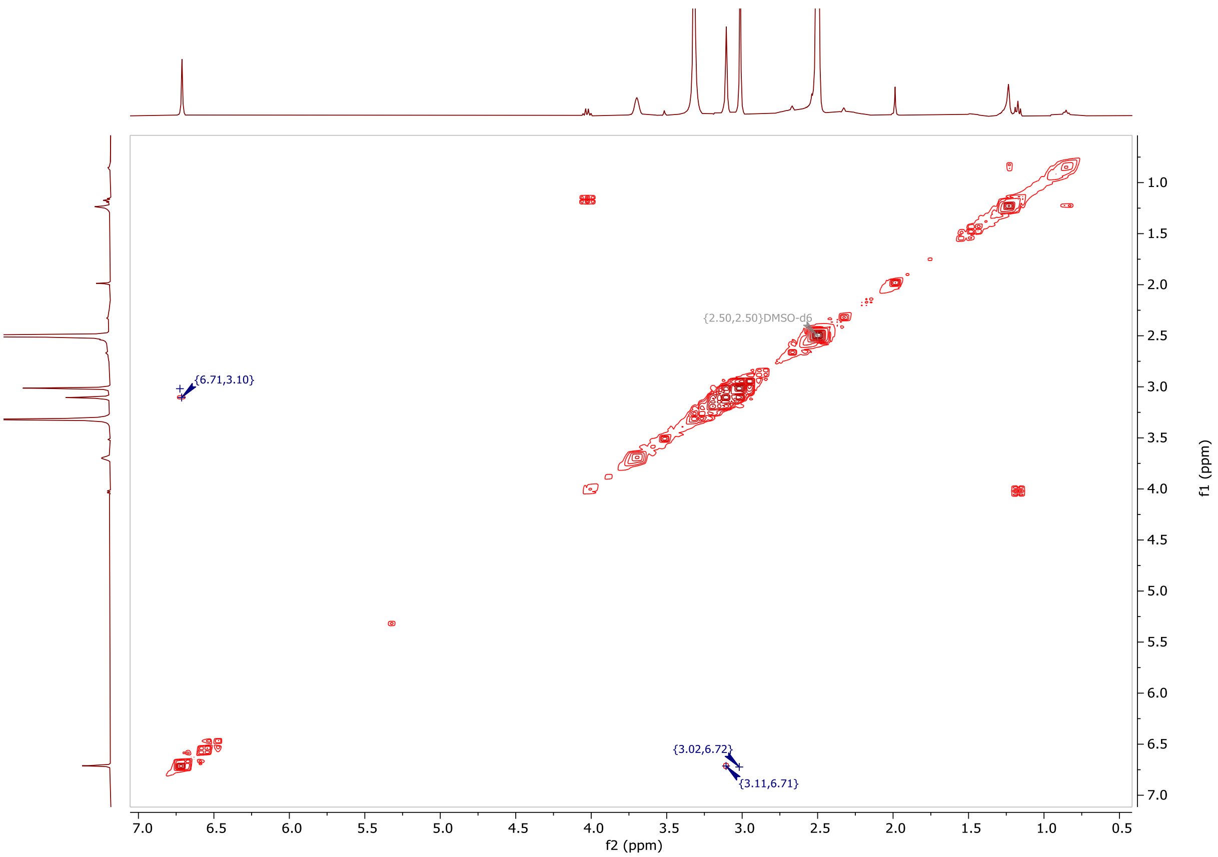


Figure S 16 H,H-COSY of **4** in $(CD_3)_2SO$

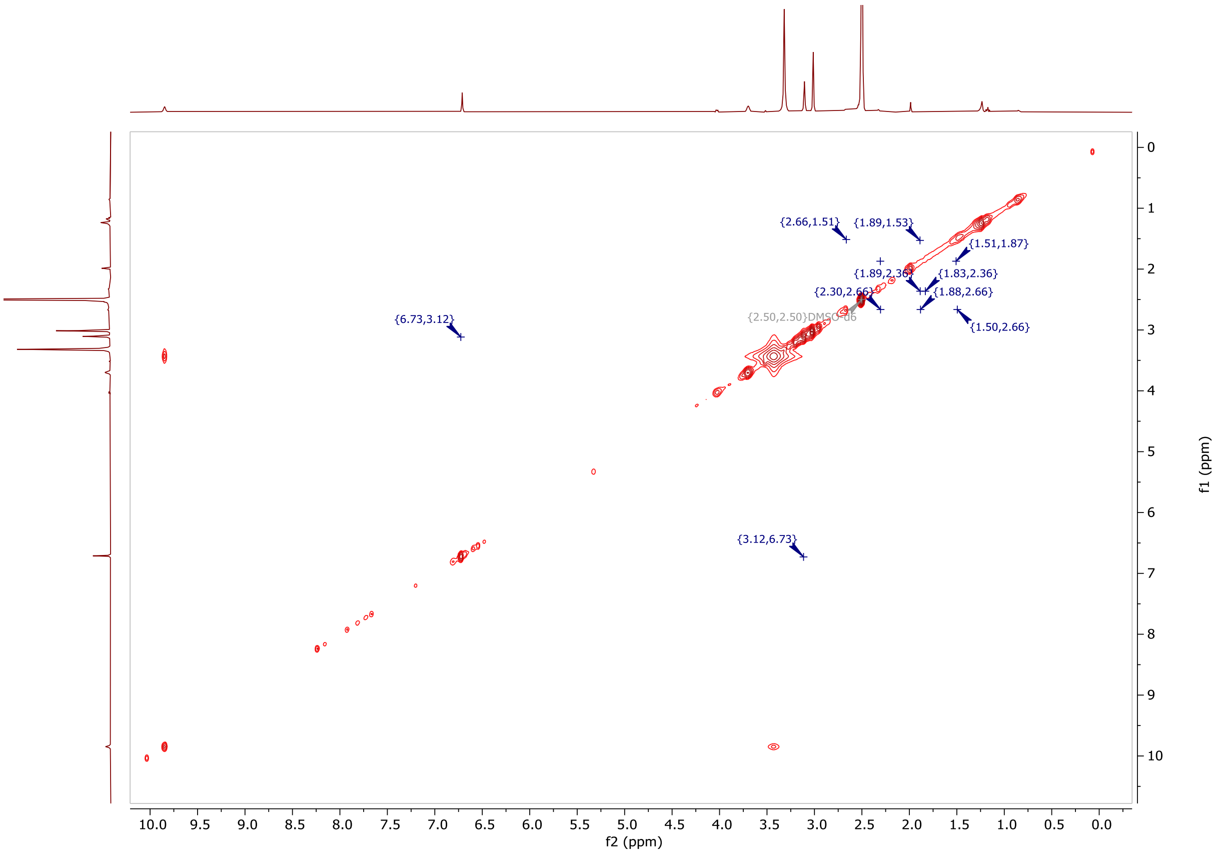


Figure S 17 NOESY of **4** in $(CD_3)_2SO$

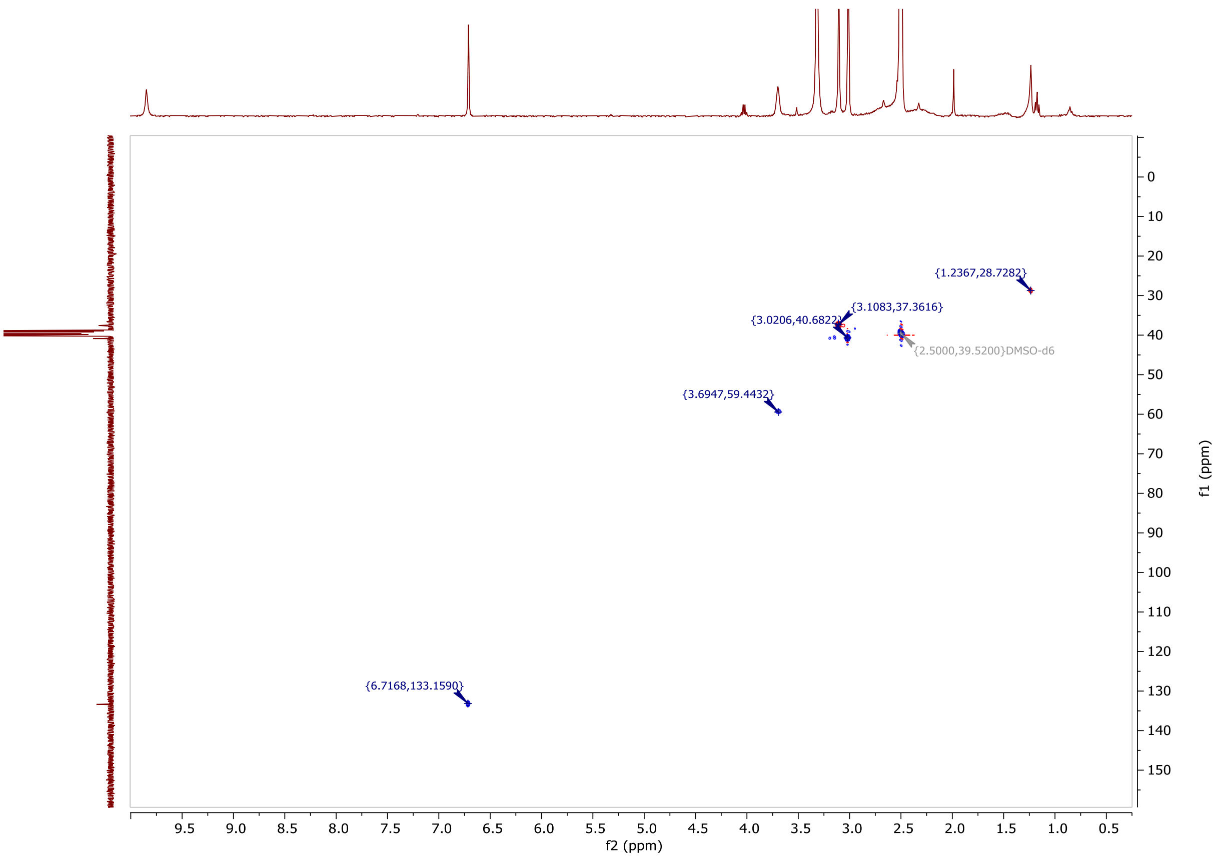


Figure S 18 HSQC of **4** in $(CD_3)_2SO$

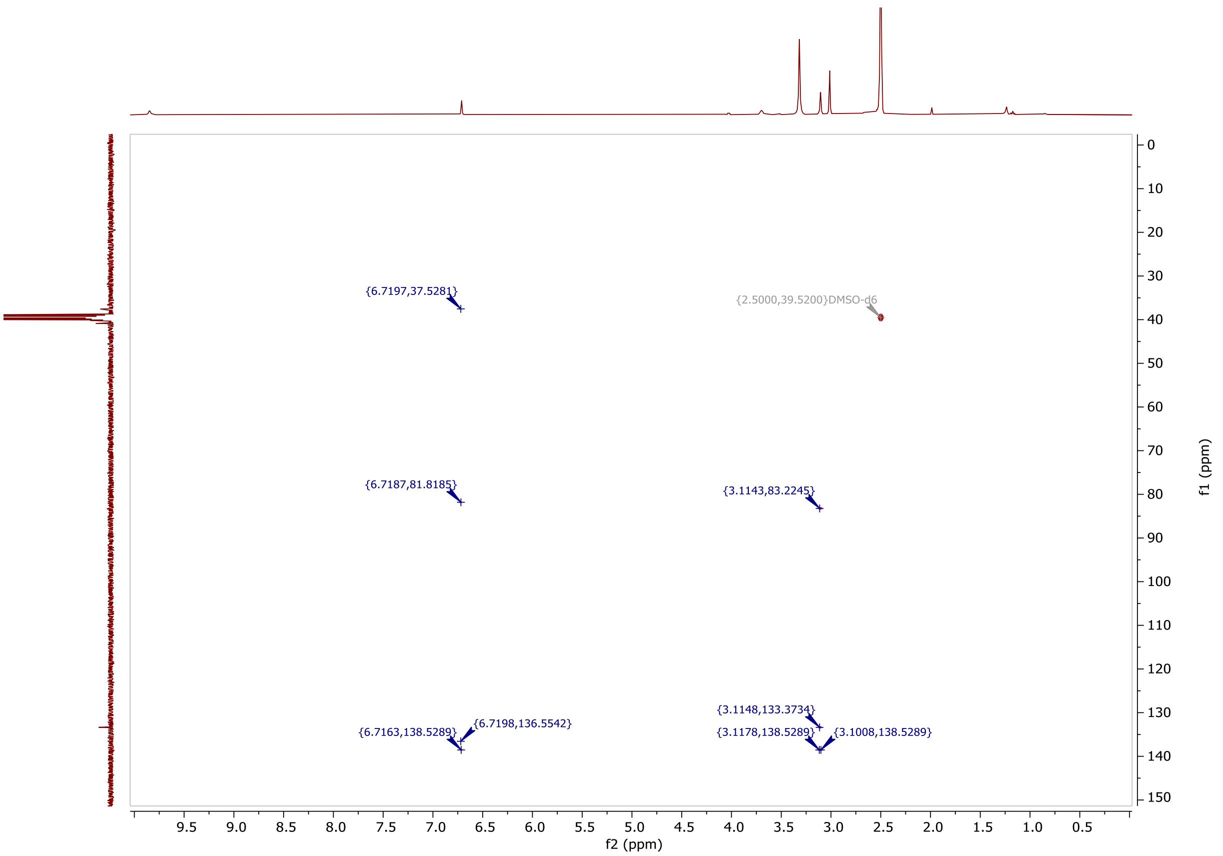


Figure S 19 HMBC of **4** in $(CD_3)_2SO$

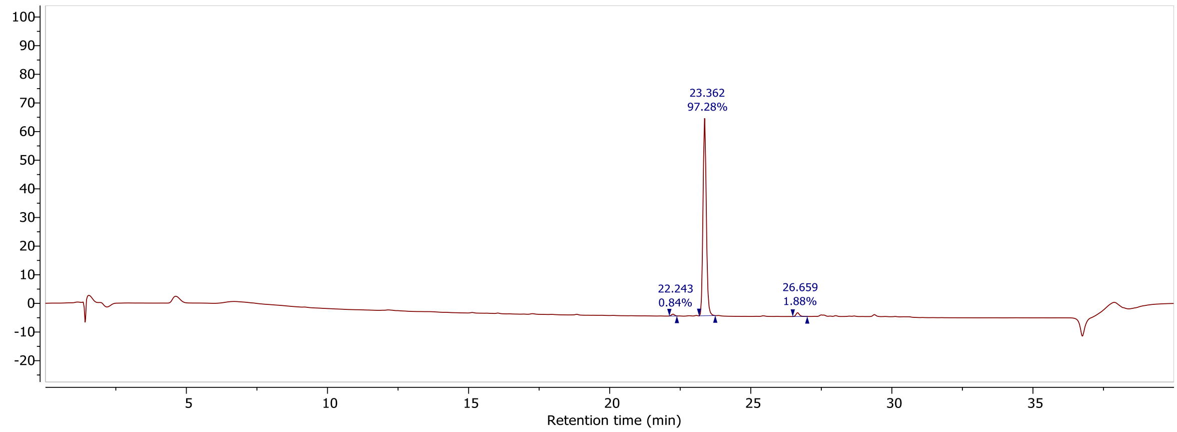


Figure S 20 HPLC purity analysis of **3** (system 5, gradient 2)

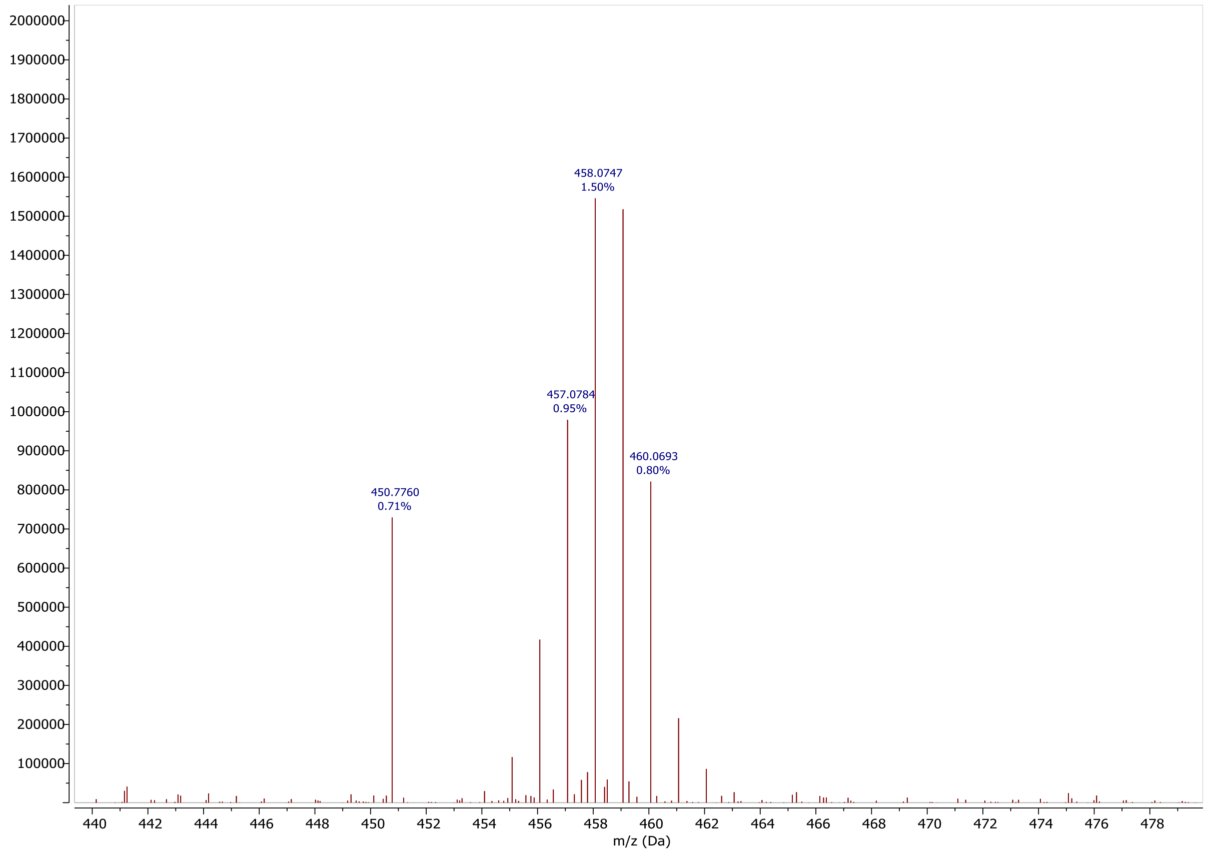


Figure S 21 HR-MS of **3** (HR-MS1, ESI$^{-}$)

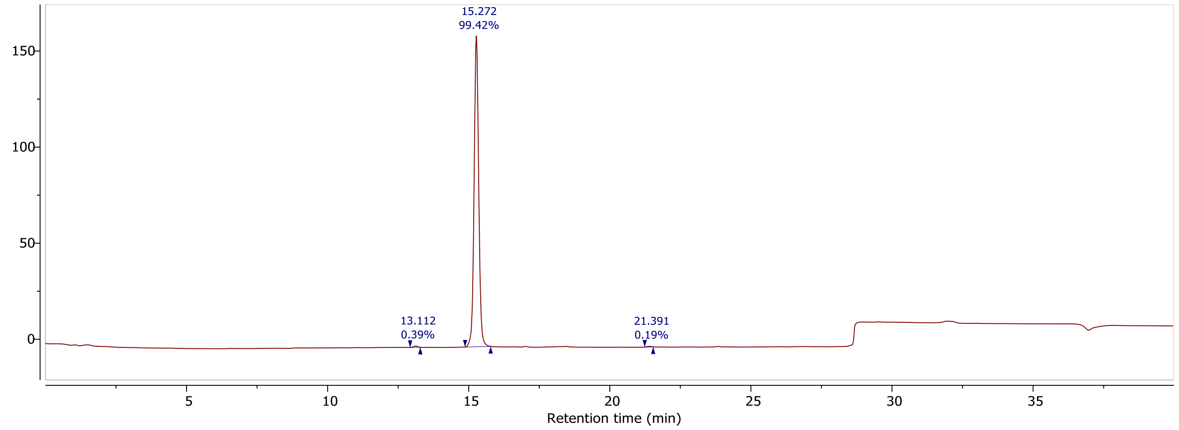


Figure S 22 HPLC purity analysis **4** (system 5, gradient 3)

# Analysis Report

Agilent

**- Scan (rt: 0.200-0.499 min) Sub  Peak 3 from - TIC Scan**

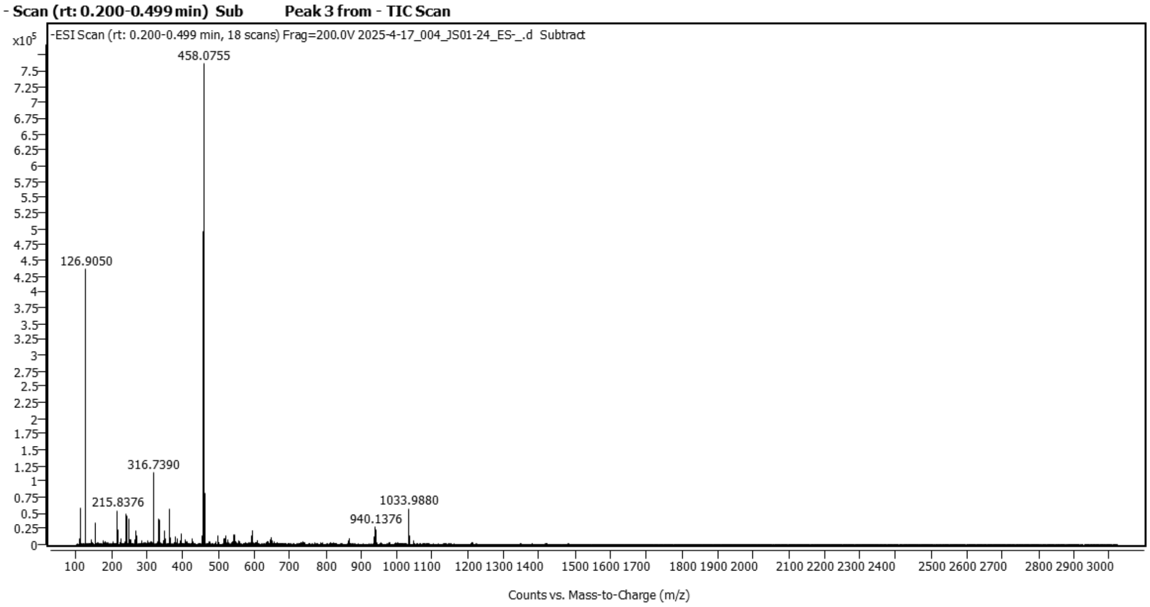


Spectrum Peaks

| m/z | Z | Abund | Abund % | m/z (Calc) | Diff (ppm) | Ion Species | Formula | Ion Type |
|---|---|---|---|---|---|---|---|---|
| 112.9855 | | 58393 | 7.66 | | | | | |
| 126.9050 | | 436130 | 57.24 | | | | | |
| 154.9737 | | 34255 | 4.50 | | | | | |
| 215.8376 | | 53163 | 6.98 | | | | | |
| 240.1731 | | 32759 | 4.30 | | | | | |
| 241.1695 | | 48729 | 6.40 | | | | | |
| 242.1661 | | 45873 | 6.02 | | | | | |
| 248.9603 | | 40129 | 5.27 | | | | | |
| 316.7390 | | 113304 | 14.87 | | | | | |
| 318.7372 | | 49546 | 6.50 | | | | | |
| 331.1823 | | 26164 | 3.43 | | | | | |
| 332.1784 | | 40775 | 5.35 | | | | | |
| 333.1753 | | 38328 | 5.03 | | | | | |
| 362.9405 | | 55956 | 7.34 | | | | | |
| 455.0862 | | 61290 | 8.04 | | | | | |
| 456.0825 | | 209525 | 27.50 | | | | | |
| 457.0790 | | 495976 | 65.10 | | | | | |
| 458.0755 | | 761872 | 100.00 | | | | | |
| 459.0721 | 1 | 713202 | 93.61 | | | | | |
| 460.0691 | 1 | 335257 | 44.00 | | | | | |
| 461.0688 | 1 | 81722 | 10.73 | | | | | |
| 462.0651 | | 27241 | 3.58 | | | | | |
| 939.1409 | | 26105 | 3.43 | | | | | |
| 940.1376 | | 27680 | 3.63 | | | | | |
| 1033.9880 | | 56739 | 7.45 | | | | | |

**- Scan (rt: 0.517-0.570 min) Sub  Peak 4 from - TIC Scan**

Figure S 23 HR-MS of **4** (HR-MS2, ESI$^-$)

# Assays

## COX Assay

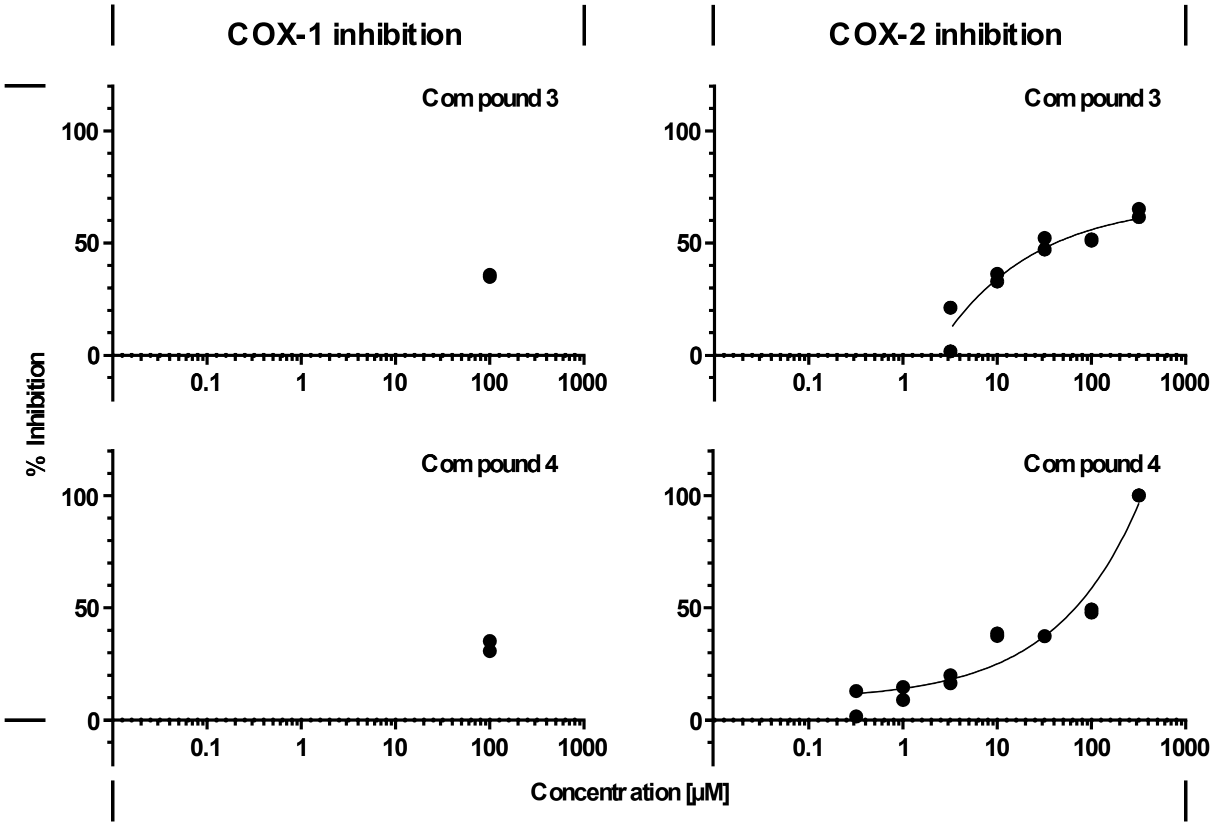


Figure S 24 COX inhibition as determined using COX Fluorescent Inhibitor Screening Assay Kit. Left column COX-1, right column COX-2. Top row **3**, bottom row **4**.

## 5-LO Assay

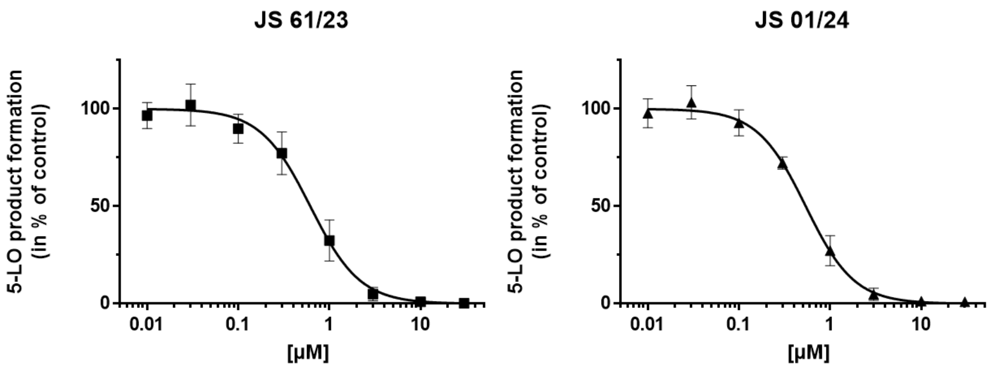


| | JS 61/23 | JS 01/24 |
|---|---|---|
| $IC_{50}$ (5-LO) | **0.62 µM**<br>(95% CI 0.53 – 0.72 µM) | **0.54 µM**<br>(95% CI 0.48 – 0.61 µM) |

Figure S 25 5-LO inhibition in whole cell PMNL assay by **3** (JS 61/23) and **4** (JS 01/24).

## Radiolabeling

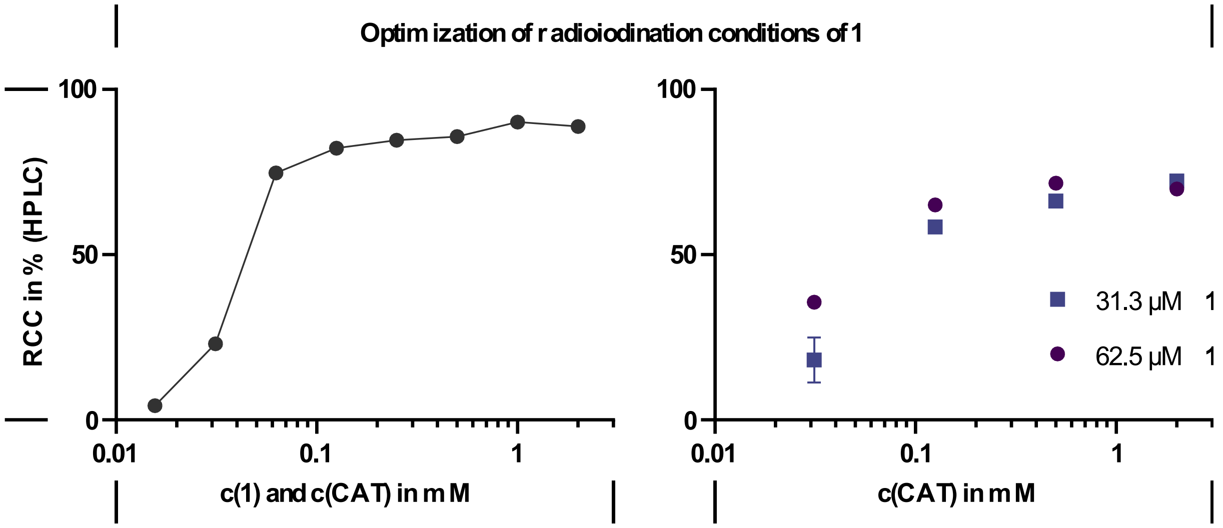


Figure S 26 Optimization of radioiodination conditions of **1** in the presence of chloramine-T (CAT). Left: successive dilution of **1** and CAT. Below 100 µM, RCC harshly decreases. Right: Increasing concentrations of CAT reestablish RCC up to 60 %, but initial values of > 80 % are not achieved.

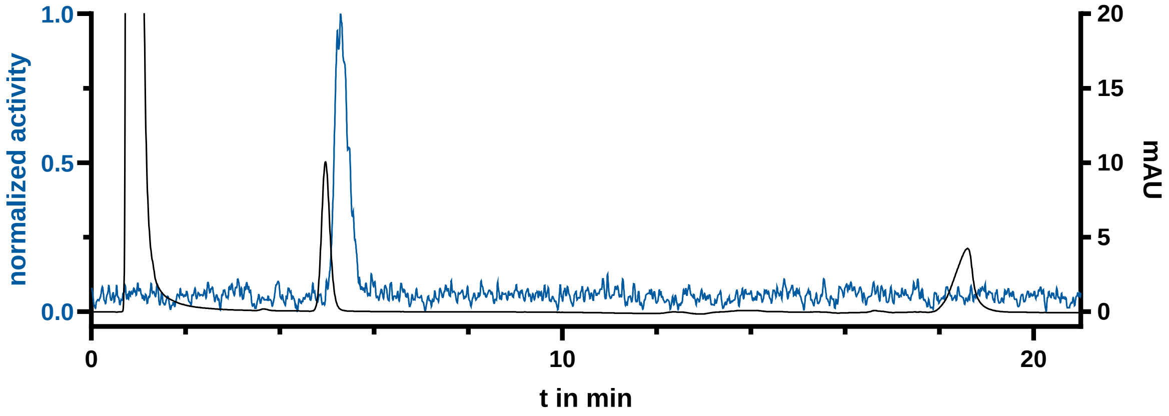


Figure S 27 **[$^{123}$I]3** coinjected with **3** (system 5, gradient 1).

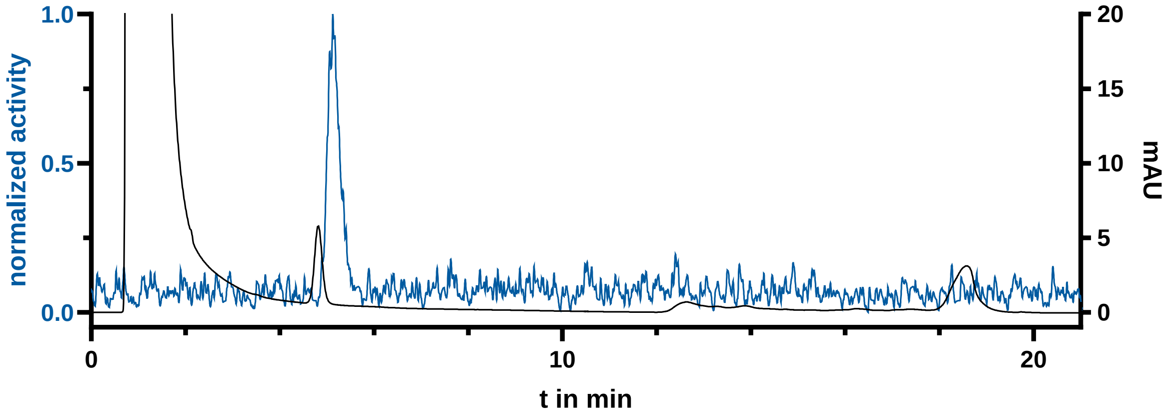


Figure S 28 **[$^{123}$I]4** coinjected with **4** (system 5, gradient 1).

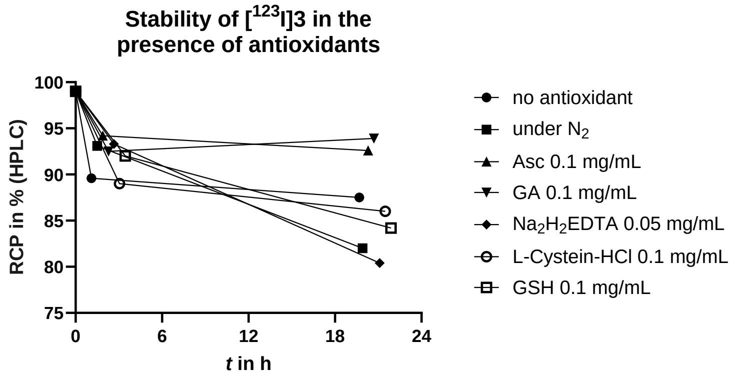


Figure S 29 Incubation of **[$^{123}$I]3** in the presence of different antioxidants.

Table S 1 RCY, RCP and $A_M$ of **[$^{123}$I]3** in the presence of antioxidants (none, Asc, GA).

| Antioxidant | Starting activity in MBq | Yield in MBq | RCY in % | RCP in % | $A_M$ in GBq/µmol |
|---|---|---|---|---|---|
| none | 56–95 | 25–40 | 53–59 ($n$ = 2) | 62–100 | n.d. |
| Asc | 102–772 | 20–406 | 41–71 ($n$ = 21) | > 87 | > 39 |
| GA | 95–690 | 59–310 | 39–67 ($n$ = 10) | > 97 | > 36 |

n.d. = not determined.

Table S 2 RCY, RCP and $A_M$ of **[$^{123}$I]4** in the presence of antioxidants (Asc, GA).

| Antioxidant | Starting activity in MBq | Yield in MBq | RCY in % | RCP in % | $A_m$ in GBq/µmol |
|---|---|---|---|---|---|
| Asc | 113–682 | 37–344 | 53–67 ($n$ = 16) | > 90 | > 171 |
| GA | 133–758 | 76–371 | 48–63 ($n$ = 3) | > 98 | > 776 |

# Stability of Radiotracers [$^{123}$I]3 and [$^{123}$I]4

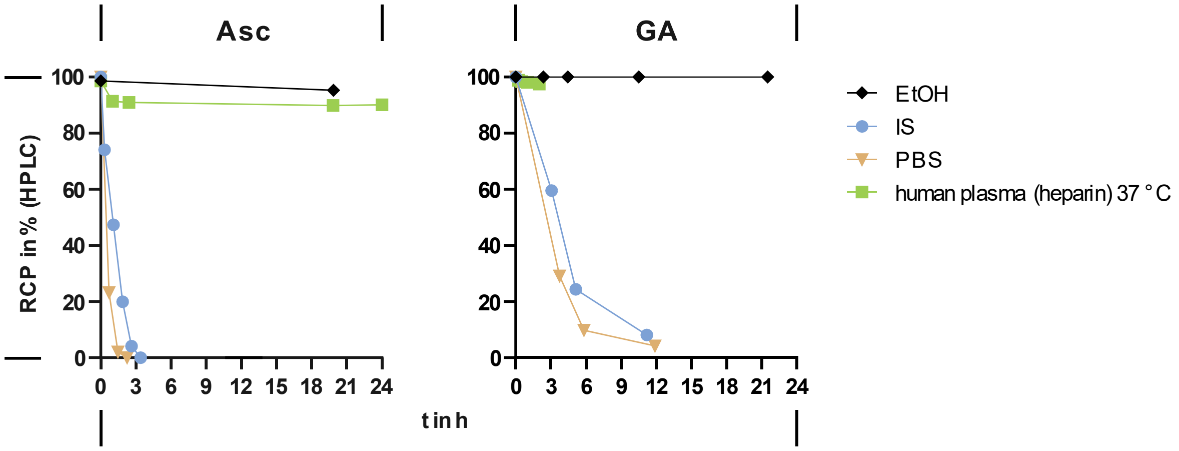


Figure S 30 Stability of **[$^{123}$I]4** in the presence of antioxidants Asc and GA.

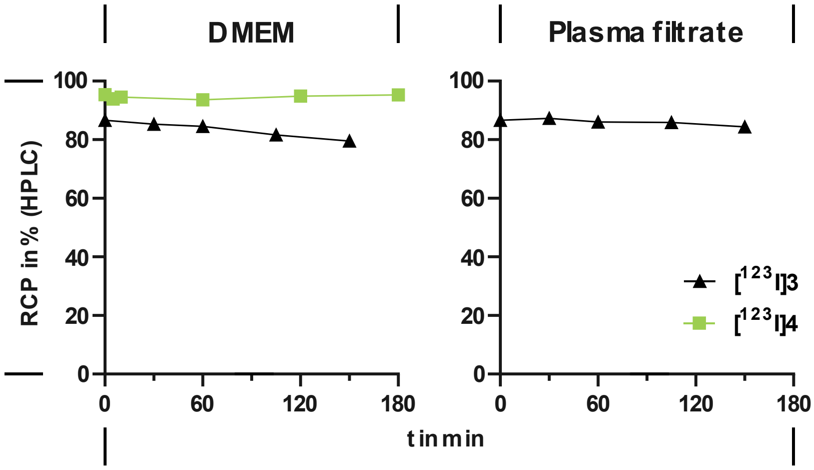


Figure S 31 Stability of **[$^{123}$I]3** and **[$^{123}$I]4** in DMEM and human plasma filtrate in the presence of Asc.

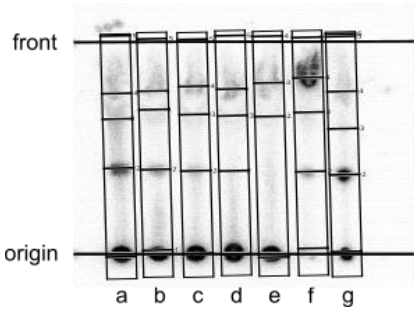


Figure S 32 Radio-TLC following murine liver microsome assay of **[$^{123}$I]3**. Samples withdrawn after 5 min (a), 10 min (b), 15 min (c), 30 min (d), and 60 min (e). Sample of tracer incubation with added non-radioactive reference substance **3** (final concentration 10 µM; f). Sample of tracer incubation under control conditions (without added NADPH; g).

# Western blot images

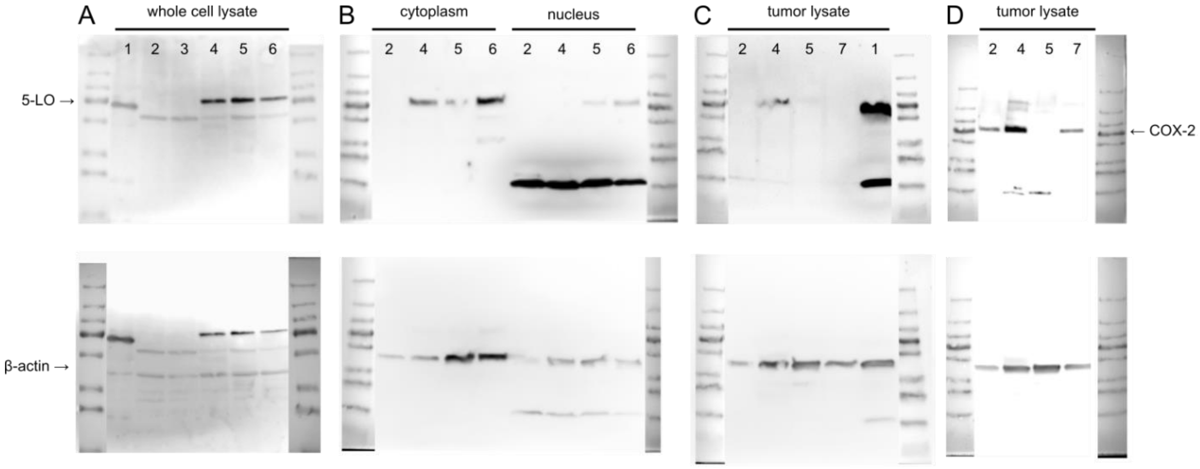


Figure S 33 Western blot analysis with 5-LO antibody (A, B, C) and COX-2 antibody (D). Bottom row: loading control with β-actin. (A) Whole cell lysates, NMRI$^{nu/nu}$ lung tissue served as positive control for 5-LO. (B) Fractionated cell lysates from cytoplasm and nucleus. (C) Tumor lysates after explantation from xenografted NMRI$^{nu/nu}$ mice with NMRI$^{nu/nu}$ lung tissue as positive control for 5-LO. (D) Tumor lysates after explantation from xenografted NMRInu/nu mice with mouse pheochromocytoma cells as positive control for COX-2. Samples are labelled as follows: (1) NMRI$^{nu/nu}$ lung tissue, (2) U87 cells or tumor, (3) U87$^{COX\text{-}2KO}$ cells, (4) HT-29 cells or tumor, (5) MC cells or tumor, (6) MΦ cells, (7) mouse pheochromocytoma cells.

# Cell uptake studies

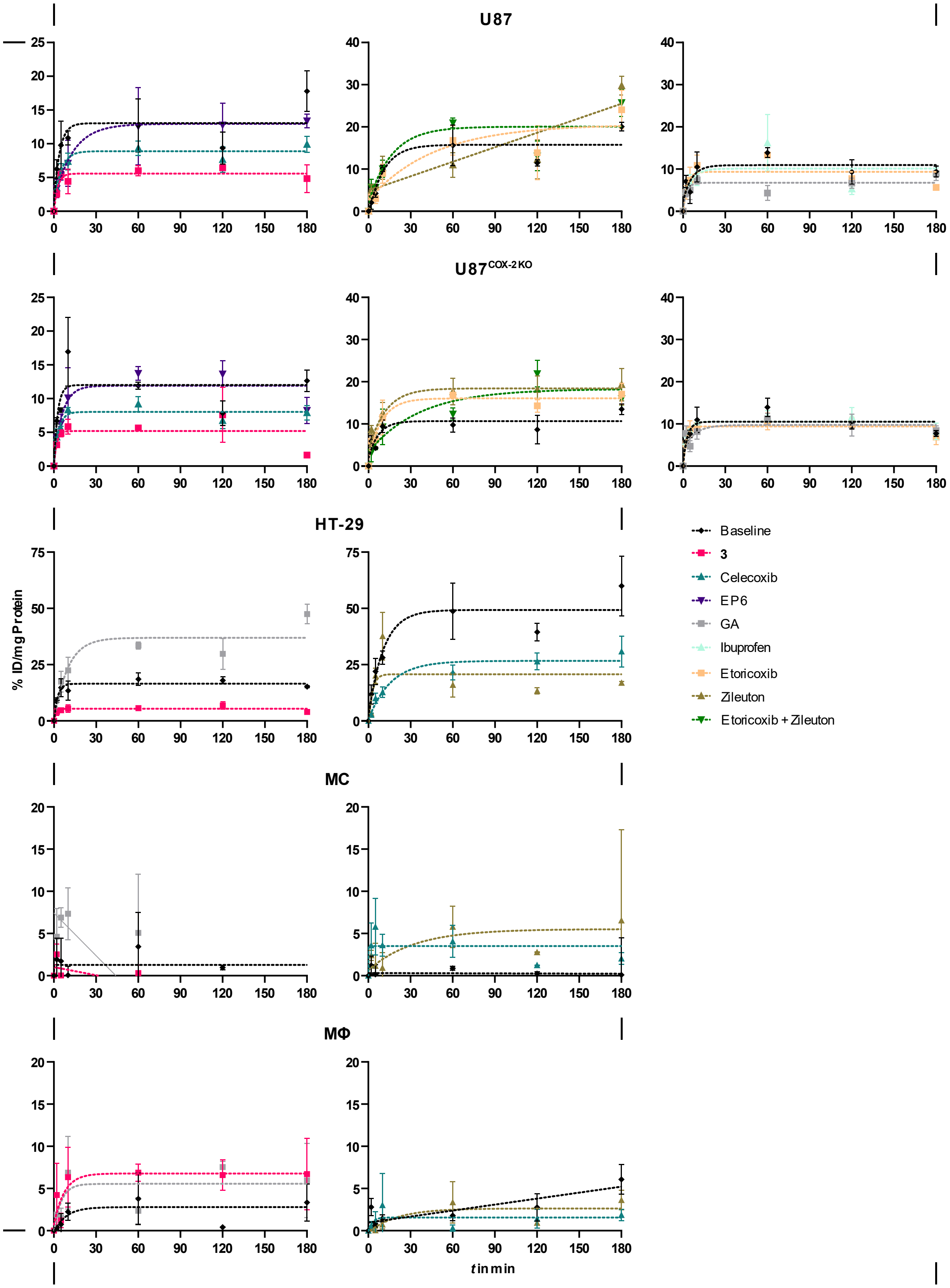


Figure S 34 Cell uptake studies of **[$^{123}$I]3**.

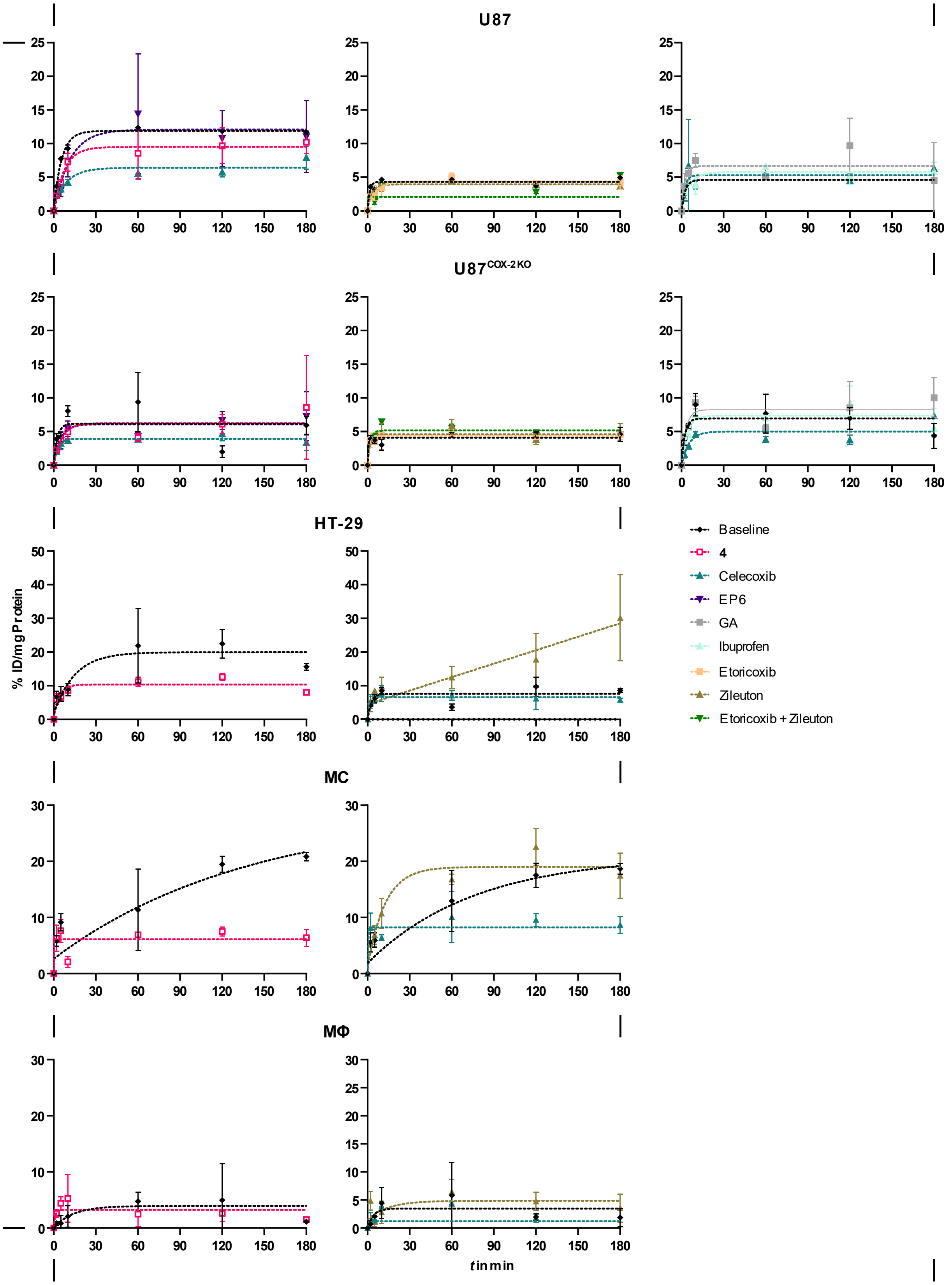


Figure S 35 Cell uptake studies of **[$^{123}$I]4**.

# SPECT images

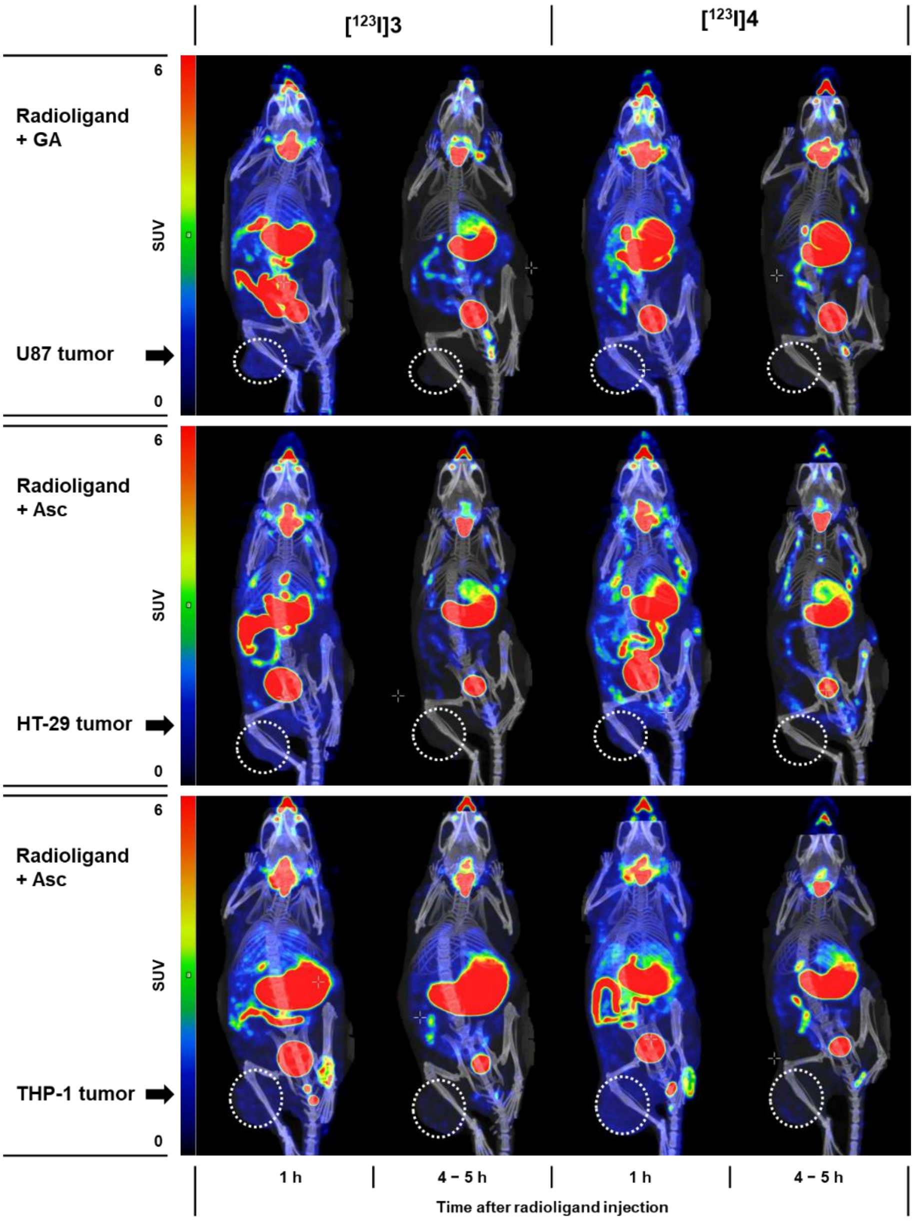


Figure S 36: Distribution of **[$^{123}$I]3** and **[$^{123}$I]4** in mice visualized using quantitative SPECT imaging. Maximum-intensity projections U87, HT-29, or THP-1 tumor-bearing mice at indicated time points after intravenous injection of the radiolabeled compounds (n.c.a), each administered at an initial dose of 20 MBq in presence of ascorbic (Asc) acid or gentisic acid (GA) as antioxidant. (SUV) standardized uptake value

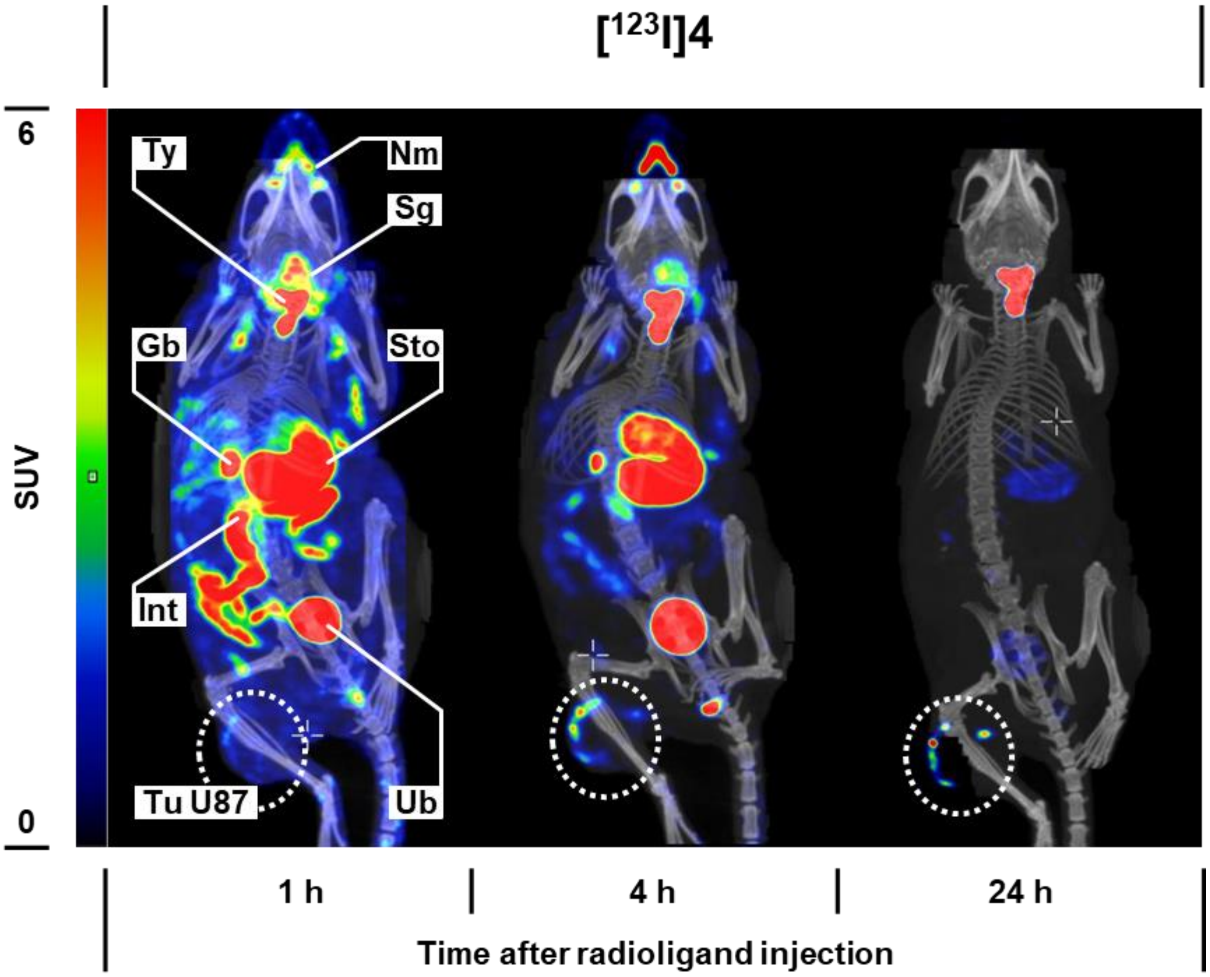


Figure S 37 Distribution of **[$^{123}$I]4** in a U87 xenografted mouse visualized using quantitative SPECT imaging as reported earlier.[116] Maximum-intensity projections at indicated time points after intravenous injection of the radiolabeled compound (n.c.a), administered at an initial dose of 20 MBq in presence of Asc as antioxidant. An accumulation of activity into the marginal region of the tumor was observed over the course of 4 h, which was retained over 24 h. This effect only occurred in this individual case and could not be replicated in subsequent experiments. The specific tumor was not explanted and therefore could not be further characterized regarding enzyme expression and cellular composition. The accumulation in the highly COX-2 expressing periphery of HCA-7 xenografts was reported for other COX-2 radiotracers.[64,125] However, because this pattern did not reappear, it is treated as an isolated, possibly non-specific event with uncertain biological relevance.

## Metabolite analysis in urine

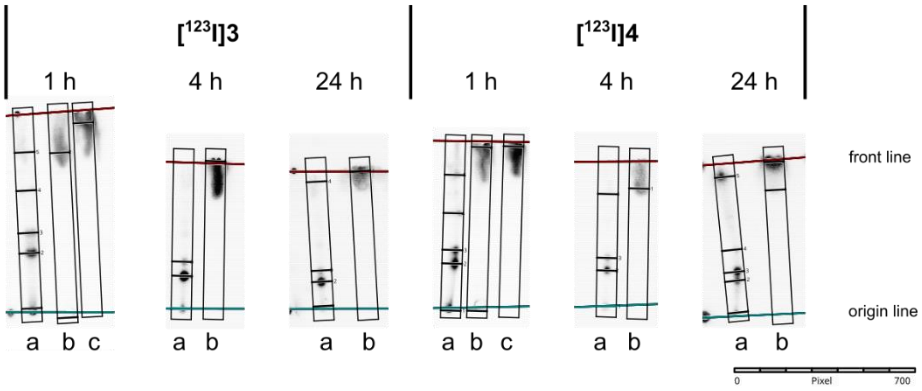


Figure S 38 Radio-TLC of urine samples collected 1.5 h, 4.5 h, and 24 h after injection of **[$^{123}$I]3** and **[$^{123}$I]4**, respectively. (a) reference compound, (b) sample after protein precipitation using 15 % v/v TCA, (c) sample without protein precipitation.
